# BDNF in Lower Brain Parts Modifies Auditory Fiber Activity to Gain Fidelity but Increases the Risk for Generation of Central Noise After Injury

**DOI:** 10.1007/s12035-015-9474-x

**Published:** 2015-10-17

**Authors:** Tetyana Chumak, Lukas Rüttiger, Sze Chim Lee, Dario Campanelli, Annalisa Zuccotti, Wibke Singer, Jiří Popelář, Katja Gutsche, Hyun-Soon Geisler, Sebastian Philipp Schraven, Mirko Jaumann, Rama Panford-Walsh, Jing Hu, Thomas Schimmang, Ulrike Zimmermann, Josef Syka, Marlies Knipper

**Affiliations:** 1 Department of Auditory Neuroscience, Institute of Experimental Medicine, Academy of Sciences of the Czech Republic, Vídeňská 1083, 142 20 Prague, Czech Republic; 2 Department of Otolaryngology, Hearing Research Centre Tübingen, Molecular Physiology of Hearing, University of Tübingen, Elfriede-Aulhorn-Str. 5, 72076 Tübingen, Germany; 3 Instituto de Biologíay Genética Molecular, Universidad de Valladolid y Consejo Superior de Investigaciones Científicas, E-47003 Valladolid, Spain; 4 Department of Otolaryngology, Plastic, Aesthetic and Reconstructive Head and Neck Surgery, Comprehensive Hearing Center, University of Würzburg, Josef-Schneider-Straße 11, 97080 Würzburg, Germany; 5 DNA Genotek Inc., 2 Beaverbrook Road, Kanata, ON K2K 1L1 Canada; 6 Centre for Integrative Neuroscience, University of Tübingen, Otfried-Müller-Straße 25, 72076 Tübingen, Germany; 7 Department of Clinical Neurobiology, University Hospital and DKFZ Heidelberg, In Neuenheimer Feld 280, 69120 Heidelberg, Germany

**Keywords:** BDNF, Central hyperactivity, High-spontaneous rate, low-threshold fibers, Homeostatic plasticity, Inferior colliculus, Sound detection threshold

## Abstract

For all sensory organs, the establishment of spatial and temporal cortical resolution is assumed to be initiated by the first sensory experience and a BDNF-dependent increase in intracortical inhibition. To address the potential of cortical BDNF for sound processing, we used mice with a conditional deletion of BDNF in which Cre expression was under the control of the Pax2 or TrkC promoter. BDNF deletion profiles between these mice differ in the organ of Corti (BDNF^*Pax2*^-KO) versus the auditory cortex and hippocampus (BDNF^*TrkC*^-KO). We demonstrate that BDNF^*Pax2*^-KO but not BDNF^*TrkC*^-KO mice exhibit reduced sound-evoked suprathreshold ABR waves at the level of the auditory nerve (wave I) and inferior colliculus (IC) (wave IV), indicating that BDNF in lower brain regions but not in the auditory cortex improves sound sensitivity during hearing onset. Extracellular recording of IC neurons of BDNF^*Pax2*^ mutant mice revealed that the reduced sensitivity of auditory fibers in these mice went hand in hand with elevated thresholds, reduced dynamic range, prolonged latency, and increased inhibitory strength in IC neurons. Reduced parvalbumin-positive contacts were found in the ascending auditory circuit, including the auditory cortex and hippocampus of BDNF^*Pax2*^-KO, but not of BDNF^*TrkC*^-KO mice. Also, BDNF^*Pax2*^-WT but not BDNF^*Pax2*^-KO mice did lose basal inhibitory strength in IC neurons after acoustic trauma. These findings suggest that BDNF in the lower parts of the auditory system drives auditory fidelity along the entire ascending pathway up to the cortex by increasing inhibitory strength in behaviorally relevant frequency regions. Fidelity and inhibitory strength can be lost following auditory nerve injury leading to diminished sensory outcome and increased central noise.

## Introduction

Brain-derived neurotrophic factor (BDNF) was initially identified as a survival factor for peripheral neurons (reviewed in [1, 2]). Increasing evidence is now emerging that BDNF acts locally as a factor that governs dendritogenesis and the spine phenotype [3–6] at certain stages of maturation [7]. Upon sensory experience in the auditory [8, 9], visual [10], somatosensory [11], and olfactory system [12], the dendritic complexity of parvalbumin (PV)-immunoreactive interneurons is particularly enhanced in response to increases in local cortical BDNF [4, 8, 11]. This process is assumed to enhance sensory resolution and acuity, and requires an adequate and timely sensory input [13–15]. The selective role of cortical BDNF for improvement of sensory resolution was questioned in the retina where BDNF was proposed as a mediator of early environmental enrichment effects on visual acuity [16]. Accordingly, blocking retinal BDNF by means of antisense oligonucleotides prior to eye opening was shown to prevent the effects on dendritic segregation of retinal ganglion cells [17] and development of retinal acuity [16] upon exposure to environmental enrichment. As constitutive BDNF knockout (KO) mice [18, 19] die too prematurely to assess the role of BDNF in the mature sensory organs, the role of peripheral versus central BDNF for sensory resolution and acuity is still obscure. Until now, it also remains unclear if and how these crucial effects of BDNF during the development of normal sensory function are linked to the complex changes of BDNF activity in mature systems, which have been described as either adaptive responses to injury [20] or nonadaptive responses in various brain disorders [21].

In rodent auditory systems, BDNF is downregulated in hair cells of the cochlea from birth onwards but remains expressed in the inner border and phalangeal cells that ensheath inner hair cells (IHCs) [22] and is upregulated with hearing onset in spiral ganglion neurons (SGNs) at the level of higher frequency cochlear turns (for a review, see [15]). While loss of BDNF in phalangeal cells does not affect hearing [23], the combined deletion of BDNF in the cochlea, the dorsal cochlear nucleus (DCN), and the inferior colliculus (IC) in BDNF^*Pax2*^-KO mice [24, 25] leads to reduced exocytosis of otherwise mature IHC synapses and to slightly diminished sound-induced auditory brainstem responses [25]. This finding suggested an unexpected role of BDNF in the lower parts of the auditory pathway for hearing sensitivity upon sensory experience. To obtain more insight into such a role of BDNF on basic sound processing, we compared sound sensitivity and noise vulnerability of BDNF^*Pax2*^-KO mice with that of mice in which BDNF is deleted more widespread throughout the auditory system including the auditory cortex using a mouse line in which *Cre* is driven by the TrkC promoter [26]. Strikingly, reduced sound sensitivity occurs only in BDNF^*Pax2*^-KO mice but not in BDNF^*TrkC*^-KO mice. This differential response is observed although both strains exhibit a similar BDNF deletion profile in the SGNs of the cochlea and in the IC, the midbrain integration center of the ascending auditory pathway [27, 28] [present study]. Results from recordings of extracellular sound-evoked responses from IC neurons in BDNF^*Pax2*^ wild-type (WT) and BDNF^*Pax2*^-KO mice and measurements of the density of inhibitory marker proteins along the auditory pathway and hippocampus suggest that BDNF in the lower parts of the auditory CNS or within the cochlea triggers basal PV inhibitory circuits to increase auditory fidelity upon sensory experience. However, this early improvement of sensitivity is accompanied by the risk to lose sensitivity and generate elevated noise levels when the BDNF-modified driving force is deteriorated after peripheral auditory nerve injury in the mature auditory system.

## Materials and Methods

### Animals

BDNF^*Pax2*^-KO mice were obtained by mating Pax2-*Cre* [24] with BDNF^*lox/lox*^ mice [29]. BDNF^*TrkC*^-KO mice were generated by mating TrkC-*Cre* [26] with BDNF^*lox/lox*^ mice. BDNF^*Pax2*^-KO mice had a deletion of BDNF in the cochlea, DCN, and IC [25]. In BDNF^*TrkC*^-KO mice, BDNF was conditionally inactivated in neurons. TrkC*-Cre* mice and Pax2-*Cre* mice were crossed with ROSA26 reporter (ROSA26R) mice as described [25]. For detection of β-galactosidase activity in the Pax2-*Cre*-ROSA26R or TrkC-*Cre*-ROSA26R mouse line, adult mice were used. β-Galactosidase activity was analyzed through X-gal staining and immunohistochemistry with anti-β-galactosidase antibody as described below. Deletion of the BDNF gene in distinct brain areas of BDNF^*Pax2*^-KO and BDNF^*TrkC*^-KO mice was verified by Northern and Western blots. Control and knockout mice of either sex were used for the experiments.

The care and use of animals was approved by either the University of Tübingen, Veterinary Care Unit and the Animal Care and Ethics Committee of the regional board of the Federal State Government of Baden-Württemberg, Germany, or by the Ethics Committee of the Institute of Experimental Medicine, Academy of Sciences of the Czech Republic, and followed the guidelines of the EU Directive 2010/63/EU for animal experiments.

### Tissue Preparation, X-gal Staining, Immunohistochemistry, and Ribbon Counts

Cochlear and brain tissues were isolated and dissected as previously described [30]. Briefly, cochleae and brains were fixed in 100 mM phosphate-buffered saline (PBS) containing 2 % paraformaldehyde and 125 mM sucrose, pH 7.4, for 2 and 48 h, respectively. Cochleae were decalcified in Rapid Bone Decalcifier (Eurobio, Les Ulis Cedex, France) followed by an overnight incubation in 25 % sucrose in Hanks’ buffered saline (HBS). Cochleae were embedded in O.C.T. compound (Miles Laboratories, Elkhart, IN, USA). Tissue samples were cryosectioned at 10 μm thickness for immunohistochemistry, mounted on Super-Frost*/plus microscope slides, and stored at −20 °C [31]. Brains were embedded after fixation in 4 % agarose, cut at 60 μm thickness with a Vibratome (Leica VT 1000S, Wetzlar, Germany), and stored in PBS at 4 °C. Brain regions were identified in accordance with the mouse atlas of Franklin and Paxinos [32]. Serial sections derived from coronal brain slices between 1.8 and 2.3 mm posterior to bregma were analyzed. For cochlear whole-mount preparations, the temporal bone was dissected on ice and immediately fixed for 2 h using 2 % paraformaldehyde in 100 mM PBS by infusion through the round and oval window. Cochlear turns were dissected and transferred to slides and attached to the surface using Cell-Tak (BD Bioscience, Heidelberg, Germany).

For X-gal staining, brain slices and isolated cochleae, slit from the apex to base, were incubated with the β-galactosidase staining solution containing 0.5 mg/ml X-gal (Sigma), 5 mM K_3_[Fe(CN)_6_], and 5 mM K_4_[Fe(CN)_6_] in PBS complemented with 20 mM MgCl_2_, 0.01 % sodium deoxycholate, and 0.02 % Nonidet-P40 overnight at 37 °C. After incubation with β-galactosidase staining solution, whole-mount preparations of the cochleae were performed. In case of a successful deletion of the floxed ROSA26R locus, the presence of β-galactosidase protein is visualized with the enzyme’s substrate (X-gal), resulting in a blue precipitate only in cells expressing *Cre* recombinase.

Immunohistochemistry was performed as described before [33, 34]. Briefly, after washing and permeabilization, tissue slices were incubated overnight with primary antibody at 4 °C. For double labeling studies, specimens were simultaneously incubated with both antibodies. Primary antibodies were detected with Cy3-conjugated (Jackson ImmunoResearch) and AlexaFluor 488-conjugated secondary antibodies (Life Technologies GmbH, Darmstadt, Germany). Slices were mounted with Vectashield mounting medium containing DAPI (Vector laboratories, Burlingame, CA, USA). Sections and whole-mount preparations were viewed using an Olympus BX61 microscope equipped with epifluorescence illumination.

For ribbon counting, image acquisition and CtBP2/RIBEYE-immunopositive spot counting were carried out as previously described [33]. Briefly, cryosectioned cochleae were imaged over a distance of 8 μm covering the entire IHC nucleus and beyond in an image-stack along the *z*-axis (*z*-stack). One z-stack consisted of about 16 layers with a *z*-increment of 0.49 μm; for each layer, one image per fluorochrome was acquired. To assess spatial protein distribution, *z*-stacks were three-dimensionally deconvoluted using the cellSens constrained iterative module with the Advanced Maximum Likelihood Estimation (ADMLE) algorithm (OSIS GmbH), Voxel Viewer, and Slice Viewer (cellSens, OSIS GmbH).

#### Antibodies

The following antibodies were used: mouse anti-β-galactosidase (Promega, Mannheim, Germany), mouse anti-parvalbumin, rabbit anti-SK2 (Sigma-Aldrich, Munich, Germany), rabbit anti-GFAP (Dako, Glostrup, Denmark), rabbit anti-parvalbumin, mouse anti-GAD67 (Merck Millipore, Darmstadt, Germany), rabbit anti-Arc, rabbit anti-VGLUT2, rabbit anti-MAP2 [35] (Synaptic Systems, Göttingen, Germany), rabbit anti-Iba1 (Wako Chemicals GmbH, Neuss, Germany), rabbit anti-BDNF (Santa Cruz Biotechnology, Heidelberg, Germany), rabbit anti-BK (Alomone Labs, Jerusalem, Israel), rabbit anti-CtBP2/RIBEYE (Cell Applications, San Diego, CA, USA), and rabbit anti-KCNQ4 [36].

#### Image Analysis

The acquired images were analyzed using the free software ImageJ (NIH, Bethesda, MD, USA) to evaluate the expression of parvalbumin and GAD67. In every picture, the background was reduced with the rolling ball algorithm, and therefore, the red, green, and blue channels have been separated. In the green channel (parvalbumin or GAD67), a threshold (with ImageJ standard parameters) has been applied and therefore the ImageJ built-in plugin, Analyze Particles, has been used to count the numbers of parvalbumin or GAD67 puncta.

### Northern Blots

Riboprobes were designed as described in [30]. Messenger RNA (mRNA) isolation was performed using the Oligotex mRNA Direct Mini Kit (Qiagen, Germany). mRNA was loaded onto a denaturing 0.8 % agarose formaldehyde gel and transferred onto a nylon membrane (Roche, Germany). The membrane was blocked and hybridized overnight at 65 °C with riboprobes for BDNF, Arg3.1, and cyclophilin. The membrane was incubated with anti-Dig-AP (Roche; 1:20,000). mRNA was detected with CDP-Star ready to use (Roche) and exposed to X-ray films.

### Western Blots

Proteins were extracted using the NucleoSpin RNA/protein kit (Macherey-Nagel, Germany) following the manufacturer’s instructions. SDS-PAGE and Western blotting were carried out using the “XCell II SureLock™ Mini-Cell and XCell II Blot Module” (Invitrogen, Germany) as previously described [37]. The blotted proteins were incubated with either rabbit polyclonal or mouse monoclonal antibodies for BDNF (Santa Cruz Biotechnology, Heidelberg, Germany), activity-regulated cytoskeletal protein (Arc) (Synaptic Systems, Göttingen, Germany), parvalbumin (Merck Millipore, Darmstadt, Germany), GAD67 (Merck Millipore, Darmstadt, Germany), or GAPDH (Abcam, Cambridge, UK).

### Noise Exposure

For acoustic trauma (AT), animals were anesthetized (75 mg/kg ketamine hydrochloride, Ketavet, Pharmacia, Pfizer, Karlsruhe, Germany; 5 mg/kg xylazine hydrochloride, Rompun 2 %, Bayer Leverkusen, Germany; in injection water to give an application volume of 5 ml/kg body weight) and exposed to intense pure tone noise (10 kHz, 116 dB sound pressure level (SPL) for 40 min) in a reverberating chamber, binaurally in an open field [25]. Control sham-exposed animals underwent the same procedure but with no traumatic sound presented (i.e., the speaker remained turned off).

### Hearing Function Evaluation

Hearing thresholds were evaluated from auditory brainstem responses (ABRs). The state of outer hair cells (OHCs) in the organ of Corti was studied by recording otoacoustic emissions. The ABR to click and pure tone stimuli and the cubic 2 × *f*1 − *f*2 distortion product of the otoacoustic emission (DPOAE) for *f*2 = 1.24 × *f*1 and L2 = L1 − 10 dB were recorded in mice aged 6–9 weeks. All physiological recordings were performed under anesthesia (for details, see above) in a soundproof chamber (IAC, Niederkrüchten, Germany) as previously described [38].

#### Auditory Brainstem Responses

ABRs, evoked by short-duration sound stimuli, represent the summed activity of neurons in distinct anatomical structures or nuclei along the ascending auditory pathway [39] and are measured by averaging the evoked electrical response recorded via subcutaneous electrodes. Briefly, ABRs were evoked by click (100 μs) or pure tone stimuli (3 ms duration, 1 ms rise/fall times, frequencies 2–45.3 kHz) of gradually increasing sound pressure in 5 dB steps of intensity. The response threshold was determined at each frequency as the minimal sound pressure evoking a noticeable potential peak in the expected time window of the recorded signal. For details, see [38].

#### Distortion Product Otoacoustic Emissions

We assessed OHC function by the growth function and the maximum response in the distortion product audiogram of the cubic DPOAE as described [34]. Frequency pairs of tones were between *f*2 = 4 kHz and *f*2 = 32 kHz.

### ABR Wave Form Analysis

ABR waveforms were analyzed for consecutive amplitude deflections (waves), with each wave consisting of a starting negative (n) peak and the following positive (p) peak. Peak amplitudes of ABR waves I and IV were extracted in the present study and defined as wave I: I_n_ − I_p_ (0.9–2 ms); wave IV: IV_n_ − IV_p_ (3.4–5.9 ms). A customized program was used to extract ABR peaks based on these definitions. ABR peak-to-peak (wave amplitude) growth functions were constructed for individual ears based on the extracted peaks for increasing stimulus levels. All ABR wave amplitude growth functions were calculated for increasing stimulus levels with reference to the ABR thresholds (from −20 to a maximum of 75 dB above threshold before noise exposure and from −20 to a maximum of 55 dB above threshold after noise exposure). For illustrative purposes, ABR wave amplitude growth functions were first linearly interpolated to the resolution of 1 data point/dB and then smoothed by a moving zero-phase Gaussian filter with a window length of 9 data points (9 dB). The ABR waveforms shown in the inset of the figures were smoothed by a moving zero-phase Gaussian filter with a window length of 5 data points (0.5 ms).

### Extracellular Recording of the Neuronal Activity in the IC

Four groups of animals were tested: control, wild-type (WTc, *n =* 4), and knockout (KOc, *n =* 4) mice and mice with auditory trauma, WTat (*n =* 5) and KOat (*n =* 5). Mice with auditory trauma were exposed to noise at the age of 9–10 weeks and extracellular recordings were performed at 13–20 weeks. The surgery and extracellular recording in the IC were performed in mice anesthetized with 35 mg/kg ketamine (Narkamon 5 %; Spofa, Prague, Czech Republic) and 6 mg/kg xylazine (Sedazine 2 %; Fort Dodge, Animal Health, Fort Dodge, Iowa) in saline via intraperitoneal injection. Body temperature was maintained with a DC-powered electric temperature-regulated pad. Recordings were carried out in a soundproof anechoic room.

#### Surgical and Recording Procedures

For access to the IC, an incision was made through the skin of the skull and underlying muscles were retracted to expose the dorsal cranium. A holder was glued to the skull. Small holes were drilled over both IC of the mouse; the animal was transported to the soundproof anechoic room and placed on a heating pad, which maintained a 37–38 °C body temperature. Neuronal activity in the IC was recorded using a 16-channel, single shank probe (NeuroNexus Technologies) with 100 μm between the electrode spots or single parylene-coated tungsten electrode (Bionic Technologies Inc). The signal obtained from the electrode was amplified 10,000 times, band-pass filtered over the range of 300 Hz to 10 kHz, and processed by a TDT System III (Tucker Davis Technologies, Alachua, FL, USA) using an RX5-2 Pentusa Base Station. The recorded data was processed and analyzed with a custom software based on MATLAB.

#### Frequency-Intensity Mapping

Using frequency-intensity mapping as described in [40], the tuning properties of individual IC neurons were evaluated. To determine the excitatory response area, pure tone bursts (100 ms in duration, 5 ms rise/fall times) with variable frequency (1/8 octave step) and intensity (5 dB step) were presented in random order.

A two-dimensional matrix was thereby obtained, with elements corresponding to the response magnitudes in the respective frequency-intensity points. The discrete point matrix was then converted to a smooth function of two variables, frequency and intensity, a process involving cubic smoothing spline interpolation. This averaging method allowed us to overcome the limited resolution of the frequency-intensity map. The resulting smooth function thereafter served as a basis for the extraction of all the parameters of interest: (i) the excitatory response threshold, the lowest stimulus intensity that excited the neuron, measured in dB SPL; (ii) the characteristic frequency (CF), the frequency with the minimal response threshold, measured in kHz; and (iii) the bandwidth of the excitatory area 10 dB above the excitatory threshold, measured by quality factor *Q*_10_. Quality factor *Q*_10_ is a common measure of frequency selectivity that is reciprocally related to the excitatory area bandwidth (the higher the *Q*_10_, the sharper the response), defined as *Q*_10_ = CF/bandwidth.

#### Two-Tone Stimulation

To detect inhibitory areas, a two-tone stimulation was employed [40]. Two-tone inhibition in the IC consists of two components: intrinsic inhibition and suppression from lower auditory centers. Local inhibition is typically characterized by the effects of off-CF tones on spontaneous activity. Also the two-tone stimulation paradigm is commonly used to evaluate the local inhibition on the level of the cochlear nucleus (CN) [41–43], the IC [44, 45], and the auditory cortex (AC) [46]. Using the two-tone stimulation, the contribution of cochlear suppression cannot be excluded in the current study. However, we conclude that central inhibition on the level of the IC mainly contributes to the neural inhibition, since the shapes of the inhibitory areas in representative neurons stimulated with two-tone stimulation corresponded well with those obtained by inhibition of spontaneous activity. Pure tone at the neuron’s CF fixed 10 dB above the threshold at CF and pure tone bursts of variable frequency and intensity, analogous to those used for the excitatory area mapping, were simultaneously presented. Similar to frequency-intensity mapping, a two-dimensional matrix was obtained. Spike rates of responses in noninhibited areas (outside the excitatory area), inhibited areas, and excited areas (20 dB above threshold) were determined. Inhibitory strength was calculated as the ratio of spike numbers per stimulus in inhibited areas to those in noninhibitory areas. Low- and high-frequency inhibition bands were analyzed separately. Neurons displaying inhibition with a strength of 20 % and higher were considered to be neurons displaying inhibition, and their numbers were compared between groups. In addition to inhibitory strength, the ratio of response magnitude in excitatory to noninhibitory areas was evaluated.

#### Intensity Coding in the IC

Neuronal responses to broadband noise (BBN) bursts of variable intensity (from 0 to 90 dB SPL, 10 dB steps, 50 repetitions) were used to construct the rate-intensity function (RIF) from which response threshold, maximal response magnitude, dynamic range, and relative initial slope of the RIF were determined [47]. From the RIF, further response parameters such as spontaneous firing rate and first-spike latency were assessed. Spontaneous firing rate of each neuron was determined from poststimulus time histograms to BBN stimulation from 200–300 ms after presentation of the stimulus 10 dB above the threshold. Minimum first-spike latency (mFSL) was evaluated from the poststimulus time histograms to BBN stimuli at 80 dB SPL, as the amount of time between the onset of sound presentation and the appearance of first spikes of neuronal responses.

### Statistical Analysis

Data are presented either as the mean ± standard deviation (SD) or standard error of the mean (SEM) for values with normal distributions or the median (Mdn) for values with nonnormal distributions. For statistical analysis, the GraphPad Prism software was used. To assess differences in mean or median values between groups, two-sided Student’s *t* tests, one-way or two-way ANOVA with Bonferroni’s multiple comparison test, Kruskal-Wallis tests with Dunn’s multiple comparison test, or chi-square tests were employed.

## Results

### Differential Deletion of BDNF in the Cochlea and Brain in Pax2-*Cre* Versus TrkC-*Cre* Mouse Lines

**Fig. 1:**
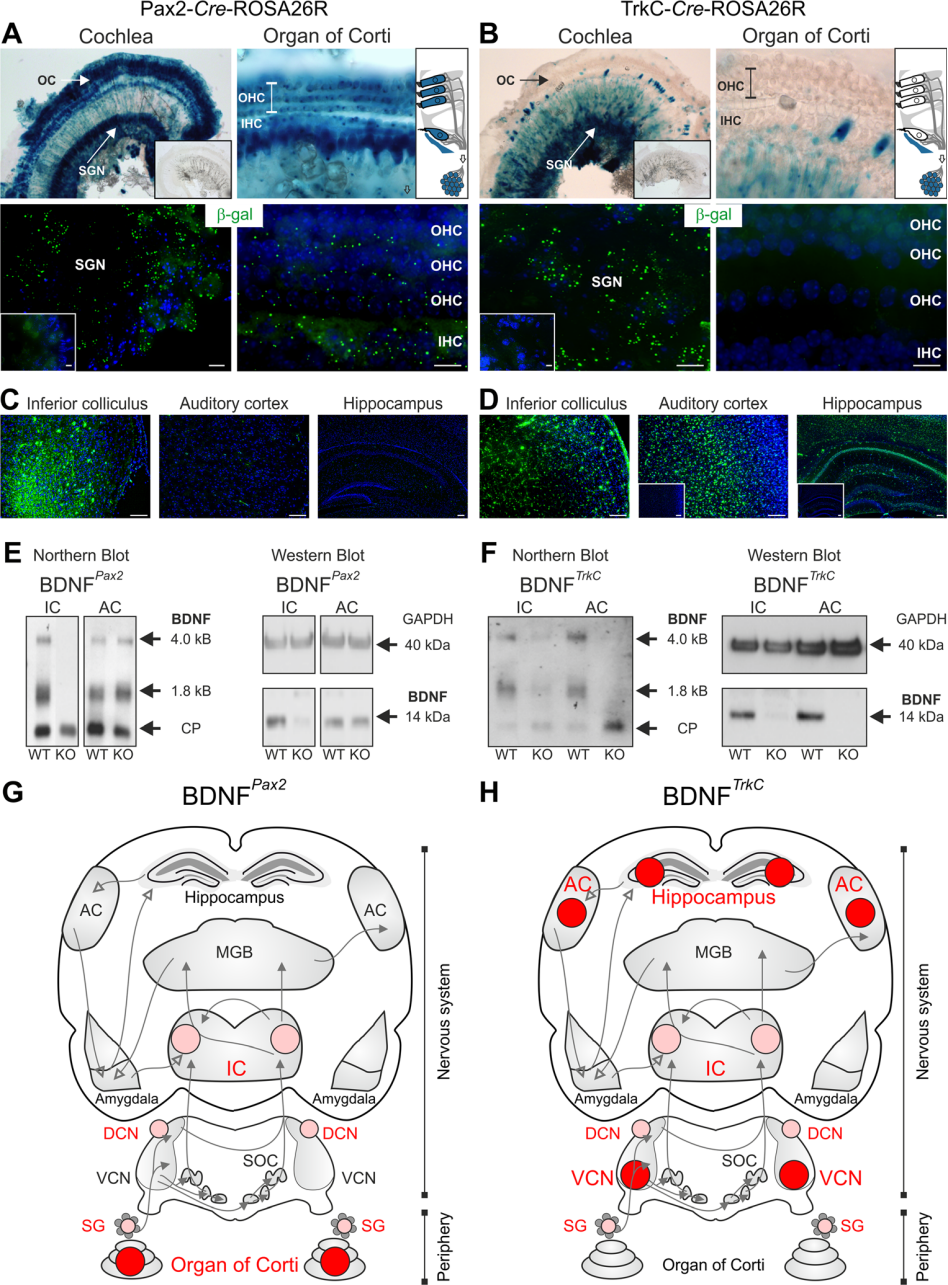
Differential BDNF deletion patterns under the Pax2 or the TrkC promoter. **a**, **b** X-gal staining and β-gal immunolabeling of mice carrying the Pax2-*Cre* (**a**) or the TrkC-*Cre* transgene (**b**) on a ROSA26R background. **a** In the mature cochlea, β-galactosidase activity in Pax2-*Cre*-ROSA26R mice was detected in inner (*IHC*) and outer hair cells (*OHC*) of the organ of Corti (*OC*) and in spiral ganglion neurons (*SGN*). **b** β-Galactosidase activity in TrkC-*Cre*-ROSA26R mice was detected mainly in SGNs, but not in hair cells, as demonstrated by immunohistochemistry. **c** Immunohistochemistry for β-gal in the inferior colliculus (*IC*), auditory cortex (*AC*), and hippocampus of Pax2-*Cre*-ROSA26R mice. In the IC, β-gal staining can be detected, whereas no expression is observed in the AC and hippocampus. **d** Immunohistochemistry for β-gal in the IC, AC, and hippocampus of TrkC-*Cre*-ROSA26R mice. Clear β-gal staining is observed in the IC, AC, and hippocampus. In *Cre*-negative mice, no β-gal staining is seen (*insets*). *Scale bars* = **a**, **b** 10 μm; **c**, **d** 100 μm. **e** Northern and Western blots from IC and AC tissues of wild-type (*WT*) and BDNF^*Pax2*^ knockout (*KO*) mice demonstrating a deletion of BDNF mRNA isoforms (1.8 and 4 kb) and BDNF protein (14 kDa) in the IC but not in the AC of BDNF^*Pax2*^-KO mice. **f** Northern and Western blots from IC and AC tissues of WT and BDNF^*TrkC*^-KO mice demonstrating a deletion of BDNF mRNA isoforms (1.8 and 4 kb) and protein (14 kDa) in the IC and AC of BDNF^*TrkC*^-KO mice. For Northern blots, cyclophilin (*CP*) was used as a reference (0.8 kb); for Western blots, GAPDH (40 kDa) was used as a loading control. **g**, **h** Diagrams of the auditory pathway: the area of BDNF deletion exclusively in either the BDNF^*Pax2*^-KO or the BDNF^*TrkC*^-KO is marked in *dark red*; the area of BDNF deletion in both BDNF-KO mouse lines is marked in *bright red*. **g** BDNF deletion in BDNF^*Pax2*^-KO mice, **h** BDNF deletion in BDNF^*TrkC*^-KO mice

To define the source of BDNF that is responsible for the absence of normal hearing thresholds of BDNF^*Pax2*^-KO animals [25], we compared the auditory phenotype of BDNF-Pax2-*Cre* transgenic mice with that of BDNF-TrkC-*Cre* mice, in which the *Cre* gene is controlled by the TrkC promoter [26]. A detailed comparative analysis of BDNF deletion patterns in the ascending auditory pathway was performed for both *Cre* mouse lines, using X-gal staining and anti-β-galactosidase (β-gal) immunolabeling (Fig. 1). X-gal staining confirmed intense labeling of hair cells and SGNs for Pax2-*Cre*-ROSA26R mice (Fig. 1a, upper panel), whereas for TrkC-*Cre*-ROSA26R mice, X-gal staining was observed mainly in SGNs but not in hair cells (Fig. 1b, upper panel). A similar result was observed when β-gal immunolabeling was used (Fig. 1a, b, lower panels). Negative controls showed no staining (Fig. 1a, b, inset).

**Fig. 2:**
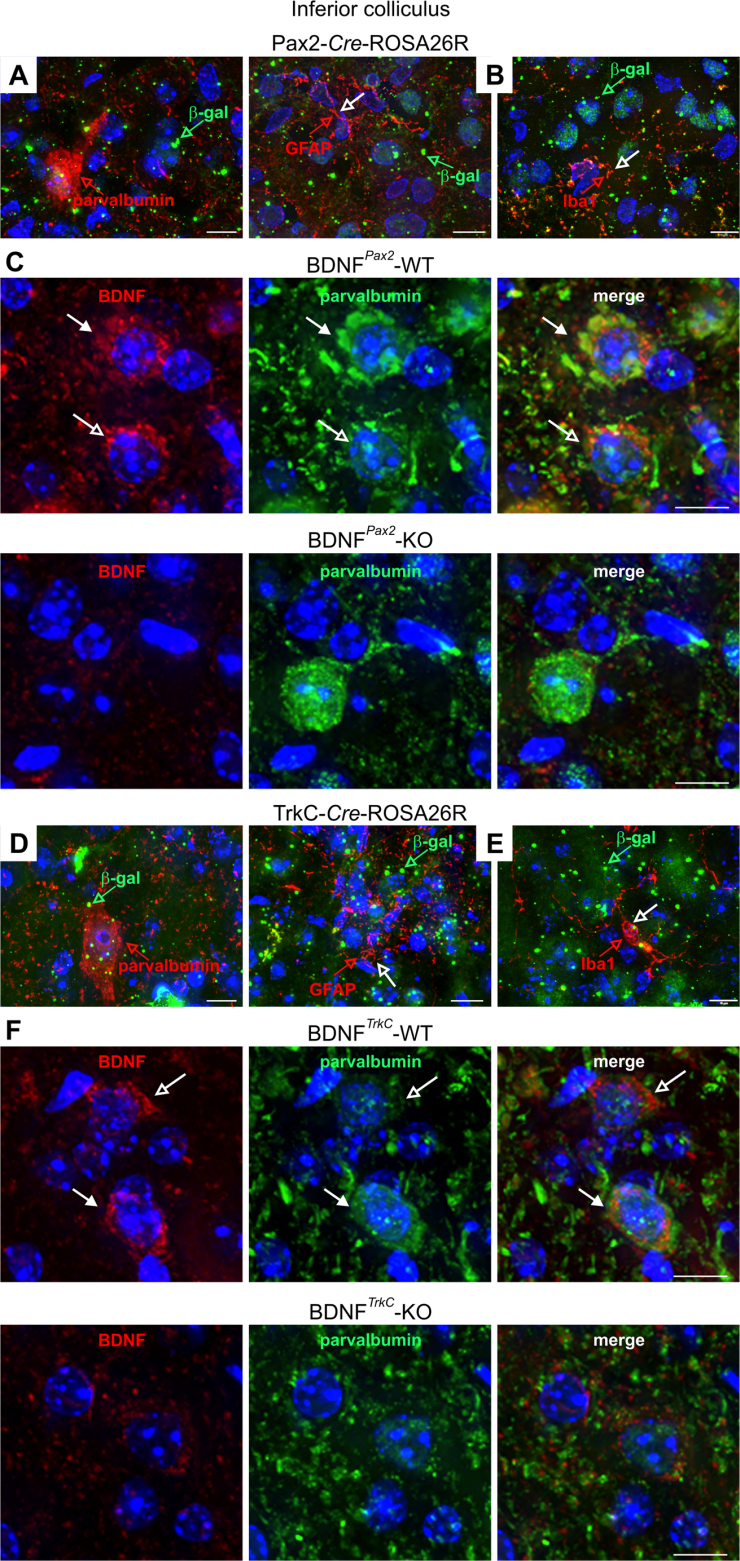
Immunohistochemistry of the IC of Pax2-*Cre*-ROSA26R and TrkC-*Cre*-ROSA26R mice and BDNF^*Pax2*^ and BDNF^*TrkC*^ WT and KO mice. **a**, **d** Immunostaining with anti-β-galactosidase (β-gal, *green*) and anti-parvalbumin (*red*) showing coexpression of β-galactosidase and parvalbumin in IC sections from both Pax2- and TrkC-*Cre*-ROSA26R mice. **b**, **e** Co-immunostaining with anti-β-galactosidase (β-gal, *green*) and either the oligodendrocyte marker anti-GFAP (*red*, *open arrow*) or the microglia marker anti-Iba1 (*red*, *open arrow*) in IC sections from both Pax2- and TrkC-*Cre*-ROSA26R mice shows no coexpression of β-galactosidase and GFAP or Iba1. Therefore, β-galactosidase is detected in neuronal cells. **c**, **f** Immunohistochemistry of IC sections stained with anti-BDNF (*red*) and anti-parvalbumin (*green*) antibodies, showing BDNF immunoreactivity in PV-positive and PV-negative neurons in BDNF^*Pax2*^-WT (**c**, *upper row*) and BDNF^*TrkC*^-WT (**f**, *upper row*) mice. *Closed arrows* indicate cells positive for BDNF (*red*) and parvalbumin (*green*). *Open arrows* indicate cells expressing only BDNF (*red*). The specificity of the BDNF antibody is shown by the lack of BDNF immunostaining in BDNF^*Pax2*^-KO and BDNF^*TrkC*^-KO mice (**c**, **f**, *lower rows*). *Scale bars* = 10 μm

At the level of the CN, β-gal was detected exclusively in the DCN in the presence of the Pax2 promoter [25], whereas with the TrkC promoter, DCN and ventral cochlear nucleus (VCN) neurons were stained (not shown). In both Pax2-*Cre*-ROSA26R and TrkC-*Cre*-ROSA26R mice, an intense and widespread staining for β-gal was observed at the level of the IC (Fig. 1c, d). In contrast, in the AC and hippocampus (Fig. 1c, d), TrkC-*Cre*-ROSA26R but not Pax2-*Cre*-ROSA26R mice exhibited β-gal staining (Fig. 1c, d). A similar deletion pattern of BDNF in both BDNF^*Pax2*^-KO and BDNF^*TrkC*^-KO mice was confirmed by the almost complete loss of the 4-kB and 1.8-kB BDNF transcripts and the absence of the 14-kDa BDNF polypeptide using Northern and Western blots, respectively (Fig. 1e, f, IC) [25]. In contrast, only in BDNF^*TrkC*^-KO but not BDNF^*Pax2*^-KO mice, BDNF mRNA and protein were absent at the level of the AC (Fig. 1e, f) [25]. The overall deletion pattern is summarized in Fig. 1g, f. In the IC, β-gal (Fig. 2a, d, green) and BDNF immunoreactivity (Fig. 2c, f, red) were found in PV-immunopositive and PV-immunonegative neurons in both BDNF^*Pax2*^-WT and BDNF^*TrkC*^-WT mice. The specificity of BDNF staining in these neurons was confirmed using BDNF^*Pax2*^-KO and BDNF^*TrkC*^-KO animals (Fig. 2c, f). Colabeling with the neuronal microtubule-associated protein MAP2 and the glutamatergic marker protein VGLUT2 indicated that PV-immunoreactive neurons in the IC may correspond to projection neurons (not shown). On the other hand, colabeling of β-gal with either the oligodendrocyte marker GFAP or the microglia marker Iba1 [48] in Pax2-*Cre*-ROSA26R and TrkC-*Cre*-ROSA26R mice (Fig. 2b, e) confirmed that β-gal was preferentially localized in the neurons of the IC.

Therefore, BDNF^*Pax2*^-KO and BDNF^*TrkC*^-KO mice exhibit comparable BDNF deletion profiles in the IC, DCN, and SGNs (Fig. 1g, h, green dots) and were expected to develop similar hearing defects.

### Deletion of BDNF in BDNF^*Pax2*^-KO but not BDNF^*TrkC*^-KO Mice Constricts Sound-Induced ABR Waves

**Fig. 3:**
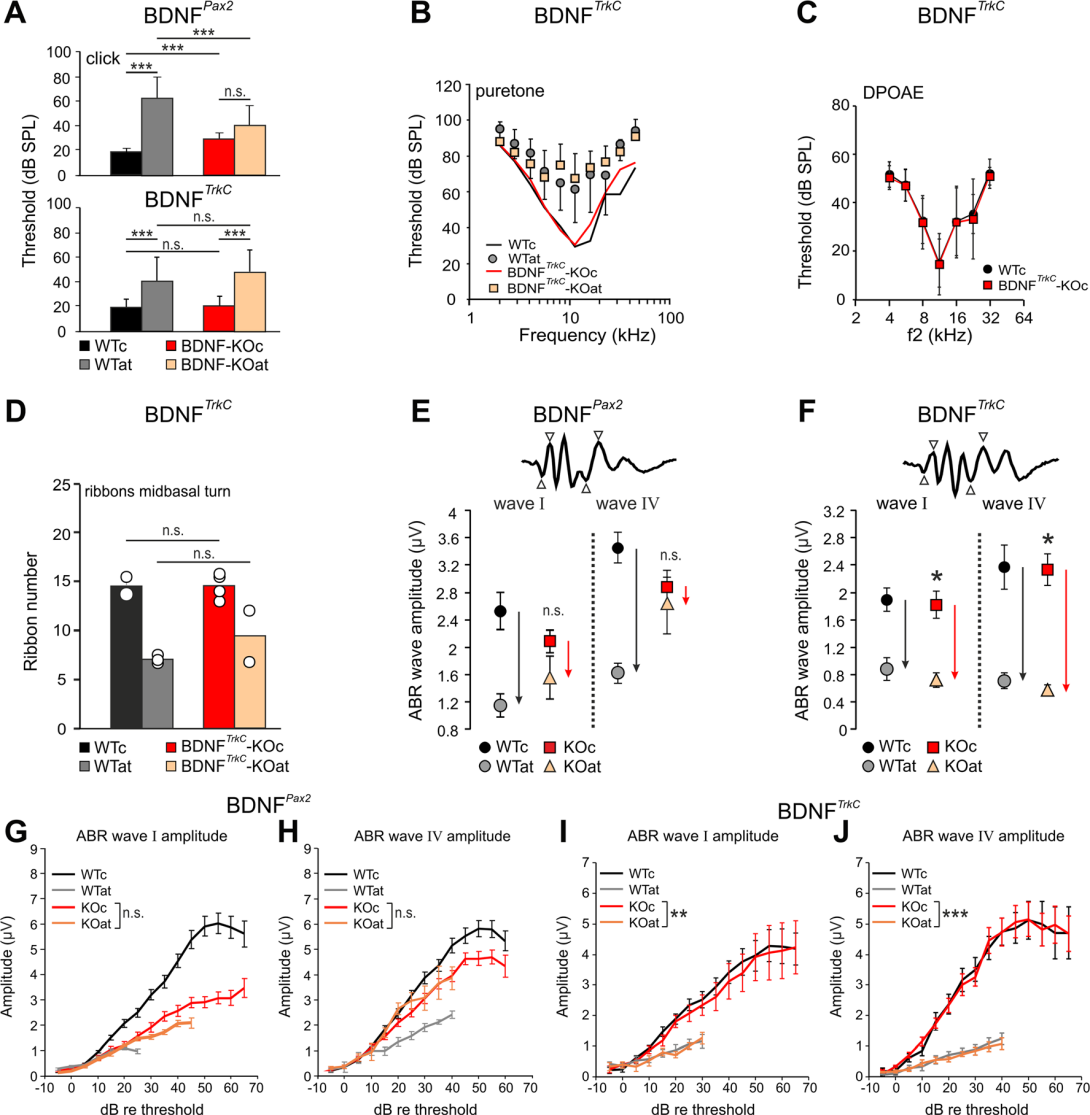
Hearing function of BDNF^*Pax2*^-KO and BDNF^*TrkC*^-KO mice before and after acoustic trauma. Auditory thresholds of BDNF^*Pax2*^-KO (**a**, *upper panel*) and BDNF^*TrkC*^-KO (**a**, *lower panel*) mice analyzed by click (**a**) and tone-burst-evoked ABR (**b**) before (KOc) and after acoustic trauma (KOat). Compared to WT mice (WTc, WTat), BDNF^*Pax2*^-KO are less vulnerable [25]. BDNF^*TrkC*^-KO mice exhibited normal hearing thresholds (**a**, *lower panel*, WTc, ears/mice: *n* = 22/11; KOc, *n* = 20/10; *p* > 0.999, two-way ANOVA) and show no significant difference to WT mice after acoustic trauma (WTat, ears/mice: *n* = 8/8; KOat, *n* = 7/7; *p* = 0.543, two-way ANOVA). *Error bars*, SD. **c** DPOAE thresholds in BDNF^*TrkC*^-WT and BDNF^*TrkC*^-KO mice were similar (WT, ears/mice: *n* = 19/10; KOn = 20/10; *p* = 0.482, two-way ANOVA). *Error bars*, SD. **d** IHC ribbon counts of midbasal cochlear turns in BDNF^*TrkC*^-WT and BDNF^*TrkC*^ *-*KO mice before (WTc, KOc) and after noise exposure (WTat, KOat). The ribbon number of BDNF^*TrkC*^-KO mice was not significantly different from that of BDNF^*TrkC*^-WT animals. *Error bars*, SEM, n.s. *p* > 0.05; WTc, sections/mice: *n* = 5/2; WTat, *n* = 6/3; KOc, *n* = 11/4; KOat, *n* = 7/2. **e**, **f** Comparison of click-evoked ABR wave amplitudes in BDNF^*Pax2*^-WT and BDNF^*Pax2*^-KO mice (**e**) and BDNF^*TrkC*^-WT (WTc, WTat) and BDNF^*TrkC*^-KO (KOc, KOat) mice (**f**) before (WTc, KOc) and after noise exposure (WTat, KOat). In BDNF^*Pax2*^-KO, suprathreshold amplitudes of wave I (auditory nerve) and wave IV (IC) are less reduced after noise exposure than in BDNF^*Pax2*^-WT mice (compare *black* and *red arrows* in **e** for different reductions in WT and KO, respectively). In BDNF^*TrkC*^-KO, the reduction was not different from the reduction in BDNF^*TrkC*^-WT mice (compare *black* and *red arrows* in **f** for similar reduction in WT and KO, respectively). Two-way ANOVA with Bonferroni’s post hoc test (**e**) wave I, n.s. *p* = 0.254; wave IV, n.s. *p* = 0.893; WTat, ears/mice: *n* = 8/4; KOat, *n* = 7/4; **f** wave I, *p = 0.04; wave IV, *p = 0.02; WTat, mice/ears: *n* = 8/16; KOat, *n* = 8/16; *error bars*, SEM. **g–j** Suprathreshold ABR amplitude at the level of the auditory nerve (wave I) and IC (wave IV). Analysis was performed before and after acoustic trauma in BDNF^*Pax2*^-WT (WTc, *n* = 16/8 ears/mice; WTat, *n* = 16/8 ears/mice) and BDNF^*Pax2*^-KO mice (KOc, *n* = 16/8 ears/mice; KOat, *n* = 16/8 ears/mice) (**g**, **h**) compared to BDNF^*TrkC*^-WT (WTc, *n* = 17/8 ears/mice; WTat, *n* = 8/4 ears/mice) and BDNF^*TrkC*^-KO mice (KOc, *n* = 15/8 ears/mice; KOat, *n* = 7/4 ears/mice) (**i**, **j**). Note the near-complete convergence of growth functions of ABR wave I (**g**, n.s. *p* = 0.275; **i**, **p = 0.008) and IV (**h**, n.s. *p* = 0.420; **j**, ***p < 0.001) before and after AT in BDNF^*Pax2*^-KO but not in BDNF^*TrkC*^-KO mice. Two-way ANOVA with Bonferroni’s post hoc test, *error bars*, SEM

We thus measured hearing function in both BDNF mutant strains as described previously [25] and were surprised to observe significant differences. As shown in Fig. 3a (upper panel), BDNF^*Pax2*^-KO mice exhibited slightly increased hearing thresholds (WTc, *n* = 6/12 ears/mice, 18.6 ± 3.92; KOc, *n* = 6/12 ears/mice, 30.3 ± 5.3; one-way ANOVA *p* < 0.001; n.s. not significant; ***p < 0.001 Bonferroni’s multiple comparison test), which have been linked with reduced exocytosis and ribbon numbers in otherwise mature IHC synapses in high-frequency cochlear turns [25]. After noise exposure, the loss of hearing thresholds in BDNF^*Pax2*^-KO animals is, however, significantly less pronounced than in BDNF^*Pax2*^-WT mice (Fig. 3a, upper panel, WTat, *n* = 8/16 ears/mice, 64.5 ± 16.64; KOat, *n* = 8/16 ears/mice, 41.9 ± 16.71; one-way ANOVA *p* < 0.001; n.s. not significant; ***p < 0.001 Bonferroni’s multiple comparison test) [25]. In contrast, BDNF^*TrkC*^-KO mice did not exhibit any differences in hearing thresholds compared to age-matched BDNF^*TrkC*^*-*WT mice, as assessed by click-evoked (Fig. 3a, lower panel, WTc, *n* = 22/11 ears/mice, 19.6 ± 6.38; KOc, *n* = 20/10 ears/mice, 20.7 ± 7.52; *p* = 0.999) and frequency-dependent (Fig. 3b, WTc, *n* = 22/11 ears/mice; KOc, *n* = 10/10 ears/mice; *p =* 0.684) hearing measurements before and after AT (Fig. 3a, WTat, *n* = 8/8 ears/mice, 40.7 ± 19.54; KOat, *n* = 7/7 ears/mice, 48.0 ± 18.03; *p* = 0.543 for click-evoked ABR, *p* = 0.419 for frequency-dependent ABR). Also, mean numbers of IHC ribbons in BDNF^*TrkC*^-KO mice before and after AT were similar to that in BDNF^*TrkC*^-WT mice (Fig. 3d). This indicates that the BDNF deletion under the Pax2 but not the TrkC promoter slightly reduced sound sensitivity.

Despite the differences in hearing thresholds, BDNF^*TrkC*^-KO and BDNF^*Pax2*^-KO mice did not differ in OHC function, based on DPOAEs (Fig. 3c, *n* = 20/10 ears/mice) [25]. Consistent with BDNF acting at the level of the IHC-afferent nerve fiber, suprathreshold amplitudes of spreading auditory nerve activity (wave I) and midbrain activity (lateral lemniscus and IC, wave IV) were significantly reduced before AT but less affected after AT in BDNF^*Pax2*^-KO compared to BDNF^*Pax2*^-WT mice (Fig. 3e, WTat, *n* = 8; KOat, *n* = 8). This effect was not observed in BDNF^*TrkC*^-KO animals (Fig. 3f, wave I: *p* = 0.885, wave IV: *p* = 0.814; WTc, *n* = 17/9 ears/mice; KOc, *n* = 15/8 ears/mice; control “c” vs. acoustic trauma “at”: WTc, *n* = 8/4 ears/mice; KOc, *n* = 8/4 ears/mice; WTat, *n* = 8/4 ears/mice; KOat, *n* = 7/4 ears/mice). Likewise, the growth of suprathreshold ABR amplitudes to increasing sound intensities was affected in BDNF^*Pax2*^-KO but not in BDNF^*TrkC*^-KO mice (compare Fig. 3g, h with i, j). Whereas ABR waves I and IV of traumatized BDNF^*Pax2*^-KO mice were significantly less elevated in comparison to those of BDNF^*Pax2*^-WT mice, no significant variations in DPOAEs (*p* = 0.056, *n* = 8/4 ears/mice) were observed after AT. Also the SPLs that generated significant differences in ABR wave I and IV 20 dB above thresholds in BDNF^*Pax2*^-WT and BDNF^*Pax2*^-KO (Fig. 3g, h) were far beyond the stimulus levels (15–30 dB SPL) where variations in the DPOAE response between BDNF^*Pax2*^-WT and BDNF^*Pax2*^-KO mice were apparent [25]. Therefore, in the range of the stimulus levels in which the ABR functions were quantified, no difference in DPOAE responses can be found between BDNF^*Pax2*^-WT and BDNF^*Pax2*^-KO animals. This indicates that the BDNF deletion under the Pax2 promoter may reduce sound sensitivity independently of OHC functions.

**Fig. 4:**
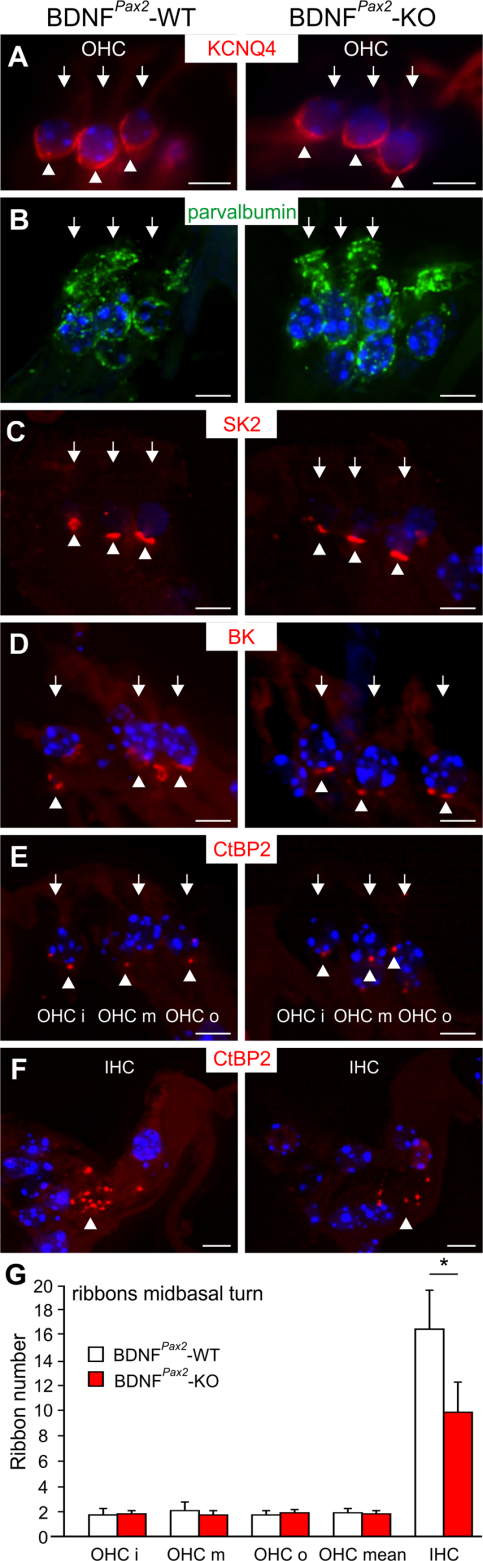
Immunohistochemistry of outer hairs cells (*OHC*, *arrows*) in BDNF^*Pax2*^-WT (*n* = 4, *left panels*) and BDNF^*Pax2*^-KO (*n* = 4, *right panels*) mice. OHCs are stained for the voltage-gated potassium channel KCNQ4 (**a**), parvalbumin (**b**), the small conductance calcium-activated potassium channel SK2 (**c**), the large-conductance calcium-activated potassium channel BK (**d**), and CtBP2, a marker of ribbon synapses (**e**). **f** In contrast to OHCs, IHCs show reduced number of CtBP2-stained ribbons in BDNF^*Pax2*^-KO mice. *Arrowheads* indicate antibody staining. **g** Quantification of ribbons stained with anti-CtBP2. No differences are observed in any row of the OHCs or in the mean ribbon number of OHCs, although a significantly reduced ribbon number can be seen in IHCs. Two-tailed unpaired Student’s *t* test with *α* = 5 (*p < 0.05). *Scale bars* = 5 μm

To further strengthen the point that the threshold differences between BDNF^*Pax2*^-WT and BDNF^*Pax2*^-KO are not caused by defects in OHC function, the phenotype of OHCs and their efferent and afferent synapses were studied in more detail. As exemplarily shown for midbasal turns, no obvious differences of the morphology of pre- and postsynapses of OHCs were observed between BDNF^*Pax2*^-WT and BDNF^*Pax2*^-KO mice. This was judged on the basis of expression of OHC marker proteins such as the outward-rectifying potassium channel KCNQ4 (Fig. 4a) or the Ca^2+^-binding protein parvalbumin (Fig. 4b). Also markers for pre- and postsynaptic efferent contacts such as SK2 and BK (Fig. 4c, d) [49, 50] and the numbers of CtBP2-stained OHC ribbons were similar between BDNF^*Pax2*^-WT and BDNF^*Pax2*^-KO mice (Fig. 4e, g), whereas IHC ribbons in the latter animals were reduced (Fig. 4f, g).

These findings suggest that BDNF deletion under the Pax2 but not the TrkC promoter reduced evoked sound amplitudes independently of OHC function. After AT, the weaker reduction of amplitudes in BDNF^*Pax2*^-KO pointed to a sound sensitivity that cannot be lost after AT, because it has never been enhanced via a sensory experience by BDNF.

The similar deletion profiles of BDNF in BDNF^*Pax2*^-KO and BDNF^*TrkC*^-KO mice shown by immunohistochemistry and Western and Northern blot analyses in the IC suggest that the IC is unlikely to be the BDNF source responsible for the altered sound sensitivity observed in BDNF^*Pax2*^-KO mice. As BDNF in the VCN or olivary complex is not deleted using the Pax2 promoter [25], the effects of BDNF acting from the superior olivary complex are also unlikely to play a role. Finally, since BDNF released from projection neurons is assumed to drive interneuron stability and not vice versa [51], loss of BDNF in PV-expressing inhibitory interneurons that are suggested to derive from Pax2 precursors [52] is probably not involved in the auditory phenotype of BDNF^*Pax2*^-KO mice. Therefore, influences from intracortically expressed BDNF acting through descending pathways are very unlikely to be the underlying cause for the auditory phenotype in the BDNF^*Pax2*^-KO animals, as in these mice BDNF is not deleted in the AC (Fig. 1c). Thus, BDNF in the lower parts of the auditory CNS or within the cochlea has to be considered as the most likely source responsible for altered sound sensitivity before and after AT in BDNF^*Pax2*^-KO mice.

### Deletion of BDNF in BDNF^*Pax2*^-KO Constricts CF Thresholds and Dynamic Range in IC Neurons

To understand the physiology behind the reduced range of suprathreshold ABR responses generated at the level of the IC in BDNF^*Pax2*^-KO mice (Fig. 3e, h), we analyzed the electrophysiological response behavior of single IC neurons in 3–4-month-old BDNF^*Pax2*^-WT and BDNF^*Pax2*^-KO mice, 3–4 weeks after sham or traumatizing acoustic exposure. The responses to BBN and pure tones of a total of 1069 IC units were recorded, comprising 264 units from WTc (*n =* 4), 232 from KOc (*n =* 4), 254 from WTat (*n =* 5), and 404 from KOat (*n =* 5).

**Fig. 5:**
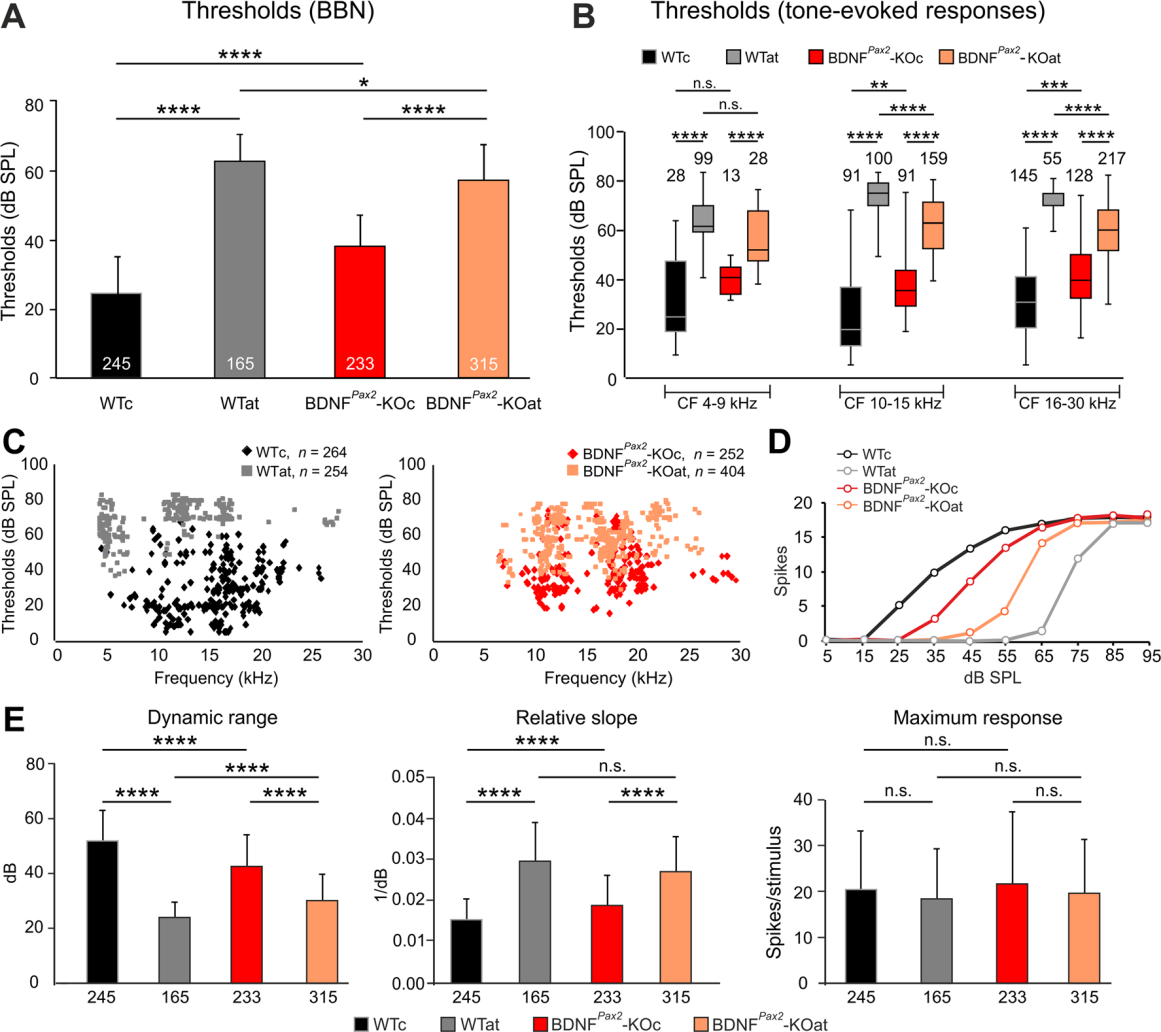
Response thresholds of IC neurons to broadband noise (*BBN*) and tone stimulation for control or sound-exposed BDNF^*Pax2*^ wild-type (*WTc*, *WTat*) and BDNF^*Pax2*^ knockout (*KOc*, *KOat*) animals. **a** Averaged thresholds of responses to BBN stimulation. **b** Box and whisker plot for thresholds of tone-evoked responses for neurons of individual CF frequency ranges (4–9, 10–15, 16–30 kHz). **c** Scatter plots of pure tone thresholds as a function of neuronal CF, shown separately for BDNF^*Pax2*^-WT (WTc, *n* = 4; WTat, *n* = 5) and BDNF^*Pax2*^-KO (KOc, *n* = 4; KOat, *n* = 5) animals. **d**, **e** Parameters of the rate-intensity function (RIF) of responses to BBN stimulation for control or sound-exposed BDNF^*Pax2*^-WT (WTc, WTat) and BDNF^*Pax2*^-KO (KOc, KOat) animals. **d** Typical examples of RIFs for all groups of animals. **e** Average dynamic range, relative slope, and maximum response for all groups of mice. One-way ANOVA with Bonferroni’s post hoc test and Kruskal-Wallis test with Dunn’s multiple comparison test, n.s. *p* > 0.05, *p < 0.05, **p < 0.01, ***p < 0.001, ****p < 0.0001. Data are presented as mean ± SD or median with interquartile range and extremes. The *numbers in the graphs* indicate the numbers of neurons

Since AT was produced by exposure to a 10-kHz pure tone, the whole sample of units was divided into three main nonoverlapping frequency bands according to their CF: 4–9 kHz (low), 10–15 kHz (middle), and 16–30 kHz (high). The thresholds of IC neurons to BBN stimulation were significantly higher in BDNF^*Pax2*^-KO than in BDNF^*Pax2*^-WT mice (Fig. 5a, BDNF^*Pax2*^-KOc, 38.62 ± 9.6 dB SPL; BDNF^*Pax2*^-WTc, 24.78 ± 10.76 dB SPL; ****p < 0.0001). After AT, in KO animals (∼20 dB), these thresholds were significantly less pronounced than in WT mice (∼40 dB; Fig. 5a, b). Also, response thresholds to tone stimulation at neuronal CF 10–15 kHz and 16–30 kHz were significantly higher in BDNF^*Pax2*^-KO than in BDNF^*Pax2*^-WT mice (Fig. 5b, middle CF, WTc: Mdn = 19.18; KOc: Mdn = 35.14, **p < 0.01; high CF, WTc: Mdn = 30.28; KOc: Mdn = 39.38, ***p < 0.001) and tended to be smaller after AT in BDNF^*Pax2*^-KO mice in these CF ranges (Fig. 5b). Low CF thresholds in BDNF^*Pax2*^-KO mice were not significantly different from WT, even though no units with thresholds below ∼30 dB SPL could be found (Fig. 5b, WTc: Mdn = 24.34; KOc: Mdn = 40.39, n.s. *p* > 0.05). Thresholds of individual IC neurons as a function of CF are presented in scatter plots for BDNF^*Pax2*^-WT and BDNF^*Pax2*^-KO animals (Fig. 5c). Finally, the parameters of the RIFs such as dynamic range (WTc 52.37 ± 11; KOc 43.23 ± 11, ****p < 0.0001) and slope (WTc 0.01424 ± 0.006; KOc 0.01919 ± 0.007, ****p < 0.0001), but not maximum response magnitude (WTc 20.52 ± 12.48; KOc 21.83 ± 15.56, n.s. *p* > 0.05) of IC neurons, were significantly reduced in BDNF^*Pax2*^-KO mice and significantly less pronounced after AT in BDNF^*Pax2*^-KO than in BDNF^*Pax2*^-WT mice (Fig. 5d, e).

These findings reveal that after the onset of hearing, BDNF in lower brain parts improves sound-evoked ABR amplitudes generated at the level of the IC (ABR wave IV amplitudes) as well as the threshold to which IC neurons can respond to sound intensities above 10 kHz. This sensitivity to sound can be lost after auditory nerve injury in the mature system only when it has been established before with the onset of hearing.

### Deletion of BDNF in BDNF^*Pax2*^-KO Mice Alters Latency and Tuning Characteristic of IC Neurons

**Fig. 6:**
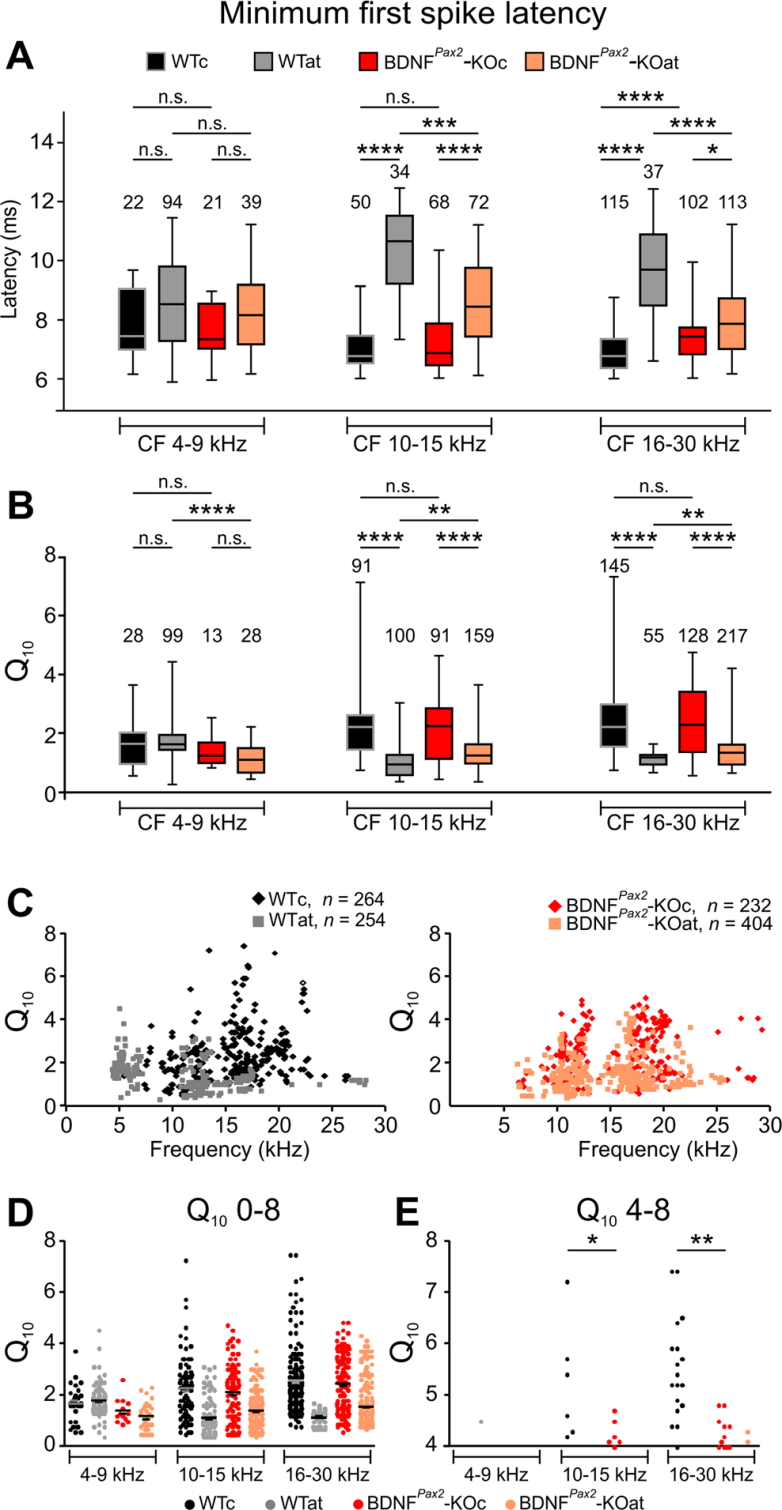
Minimum first spike latency (mFSL) and quality factor (*Q*_10_) of IC neurons. **a** mFSL of responses to 80 dB SPL BBN bursts for control and sound-exposed BDNF^*Pax2*^-WT (WTc, *n* = 4; WTat, *n* = 5) and BDNF^*Pax2*^-KO (KOc, *n* = 4; KOat, *n* = 5) animals. Box and whisker plot for mFSL for neurons of individual CF frequency ranges (4–9, 10–15, 16–30 kHz). Kruskal-Wallis test with Dunn’s post hoc test: n.s. *p* > 0.05, *p < 0.05, ***p < 0.001, ****p < 0.0001. Data are presented as medians with interquartile ranges and extremes. **b**–**e** *Q*_10_ of IC neuron responses to pure tone stimulation for control and sound-exposed BDNF^*Pax2*^-WT (WTc, *n* = 4; WTat, *n* = 5) and BDNF^*Pax2*^-KO (KOc, *n* = 4; KOat, *n* = 5) animals. **b** Box and whisker plot for *Q*_10_ for neurons of individual CF frequency ranges (4–9, 10–15, 16–30 kHz). **c** Scatter plots illustrating the *Q*_10_ as a function of neuronal CF, shown separately for BDNF^*Pax2*^-WT and BDNF^*Pax2*^-KO animals*.* **d**, **e** Scatter plots for *Q*_10_ with *y* scale 0–8 (**d**) and scale 4–8 (**e**) for the neurons of individual frequency ranges (4–9, 10–15, 16–30 kHz). Note that IC neurons >10 kHz with *Q*_10_ >5 are absent in BDNF^*Pax2*^-KO mice (**e**). Kruskal-Wallis test with Dunn’s multiple comparison test: n.s. *p* > 0.05, **p < 0.01, ****p < 0.0001. Data are presented as medians with interquartile ranges and extremes. The *numbers in the graphs* indicate the numbers of neurons

CF detection thresholds have been hypothesized to be influenced by onset stimuli [53]. Differences in onset stimuli should be reflected by differences in mFSL, which can be evaluated from poststimulus time histograms. mFSLs for 80 dB SPL stimuli were similar in BDNF^*Pax2*^-WT and BDNF^*Pax2*^-KO animals for the low- and middle-frequency bands (Fig. 6a; low CF, 4–9 kHz, WTc: Mdn = 7.28; KOc: Mdn = 7.4, n.s. *p* > 0.05; middle CF, 10–15 kHz, WTc: Mdn = 6.8; KOc: Mdn = 6.9, n.s. *p* > 0.05), but were significantly longer for neurons with high CF (16–30 kHz) (Fig. 6a; WTc: Mdn = 6.8; KOc: Mdn = 7.5, ****p < 0.0001). After AT, mFSLs were less enlarged in BDNF^*Pax2*^-KO mice for neurons with both middle and high CF (Fig. 6a, middle CF, WTat: Mdn = 10.7; KOat: Mdn = 8.5, ***p < 0.001; high CF, WTat: Mdn = 9.8; KOat: Mdn = 7.9, ****p < 0.0001). This indicates that after the onset of hearing, BDNF improves the short latency responses of IC neurons to sound. Only when these responses have been developed, such as in BDNF^*Pax2*^-WT mice, this faster response behavior can be lost after traumatizing noise in the mature system.

The quality factor *Q*_10_ of sound responses of IC neurons, which is reciprocally related to the excitatory area bandwidth 10 dB above threshold (the higher the *Q*_10_, the sharper the tuning), may help to identify the type of neuron which responds to BDNF in the IC. *Q*_10_ did not significantly differ between BDNF^*Pax2*^-WTc and BDNF^*Pax2*^-KOc mice over all CF ranges (Fig. 6b). However, after AT, *Q*_10_ in neurons with middle and high CF in BDNF^*Pax2*^-WTat mice was significantly more reduced than in BDNF^*Pax2*^-KOat animals (Fig. 6b, n.s. *p* > 0.05, **p < 0.01, ****p < 0.0001). Differences in peak *Q*_10_ values in scatter plots categorized for frequency bands in control and KO animals (Fig. 6c, d) prompted us to investigate individual *Q*_10_ values. Interestingly, in CF regions >10 kHz, the number of IC neurons with *Q*_10_ values >4 was significantly lower in sham-exposed BDNF^*Pax2*^-KO than in sham-exposed BDNF^*Pax2*^-WT animals (Fig. 6e, *p < 0.05, **p < 0.01). This indicates that after the onset of hearing, BDNF in lower brain parts sharpens a very narrow tuning characteristic of IC neurons. Only when this sharpening has developed (e.g., in BDNF^*Pax2*^-WT) it can be lost after traumatizing noise in the mature system.

### Deletion of BDNF in BDNF^*Pax2*^-KO Mice Affects the Inhibitory Strength of IC Neurons

**Fig. 7:**
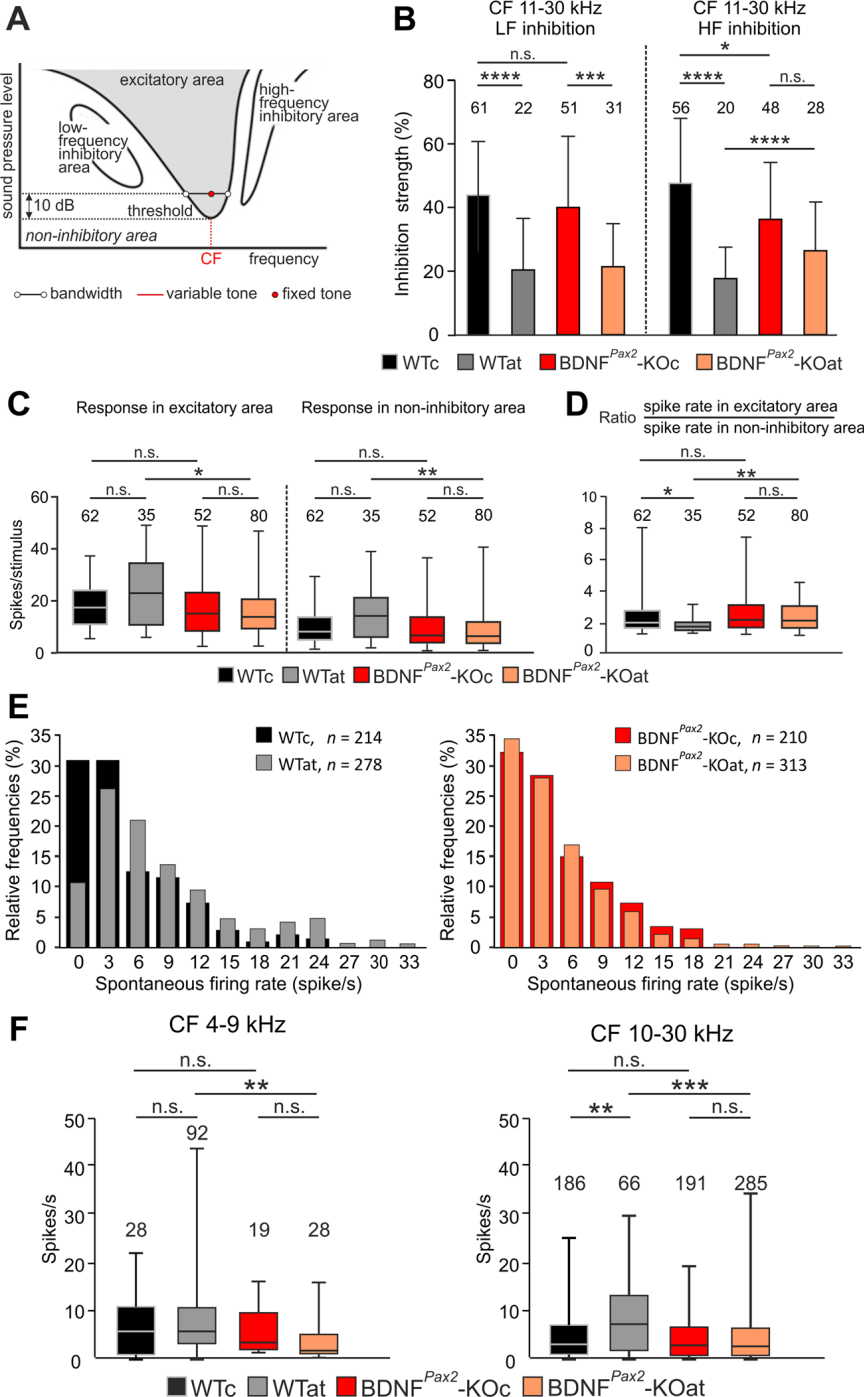
Inhibition characteristics in control or sound-exposed BDNF^*Pax2*^-WT (WTc, *n* = 4; WTat, *n* = 5) and BDNF^*Pax2*^-KO (KOc, *n* = 4; KOat, *n* = 5) animals. **a** Schematic of response map to two-tone stimulation showing excitatory, inhibitory, and noninhibitory areas. **b** Comparison of low- and high-frequency sideband inhibition strength in middle and high CF (11–30 kHz) IC neurons with inhibitory strength of 1 % and higher. One-way ANOVA with Bonferroni’s post hoc test. **c** Spike rates of IC neurons with middle and high CF in response to two-tone stimulation, determined 20 dB above threshold in the excitatory area and noninhibitory area. *Numbers in the graph* indicate number of neurons. **d** Ratio of spike rates in the excitatory field 20 dB above threshold to spike rates in the noninhibitory area. The numbers of neurons are given above the corresponding boxes. Kruskal-Wallis test with Dunn’s multiple comparison test: n.s. *p* > 0.05, *p < 0.05, ***p < 0.001, ****p < 0.0001. Data are presented as mean ± SD or median with interquartile range and extremes. **e** Distribution of IC neurons according to their spontaneous firing rate (spikes/s) for control and sound-exposed BDNF^*Pax2*^-WT (WTc, *n* = 4; WTat, *n* = 5) and BDNF^*Pax2*^-KO (KOc, *n* = 4; KOat, *n* = 5) animals. **f** Spontaneous firing rates for neurons with CF in the high-frequency band (10–30 kHz) were significantly higher in BDNF^*Pax2*^-WTat than in BDNF^*Pax2*^-WTc but were similar in BDNF^*Pax2*^-KOat and BDNF^*Pax2*^-KOc. Kruskal-Wallis test with Dunn’s post hoc test demonstrated significant differences between WTc and WTat (**p < 0.01) and WTat and KOat (***p < 0.001) groups of animals. Data are presented as mean ± SD or median with interquartile range and extremes. The *numbers in the graphs* indicate the numbers of neurons

To investigate the difference in tuning characteristics of IC neurons in more detail, inhibitory, excitatory, and noninhibitory (Fig. 7) response characteristics of IC neurons of sham-exposed and noise-exposed BDNF^*Pax2*^-WT and BDNF^*Pax2*^-KO mice were investigated using two-tone inhibition. In both groups of sham-exposed mice almost 80 % of neurons displayed low-frequency and/or high-frequency lateral inhibition. Likewise, after intense noise exposure, there was a likewise drop in the number of neurons displaying lateral inhibition in both groups of animals (data not shown).

Next, the strength of firing rate suppression by lateral inhibition was calculated as a percentage of rate suppression separately for low- and high-frequency sideband inhibition for neurons with a CF <10 kHz and CF >10 kHz. Low CF neurons did not exhibit altered low- or high-frequency sideband inhibitory strengths in the absence of BDNF or after AT (Fig. 7b). Likewise, in the neurons with a CF of 11–30 kHz, the low-frequency sideband was similar in BDNF^*Pax2*^-WT and BDNF^*Pax2*^-KO animals (Fig. 7b). However, high-frequency sideband inhibition strength was significantly reduced in sham-exposed BDNF^*Pax2*^-KO mice (Fig. 7b, *p < 0.05). Moreover, the pronounced drop in the strength of high-frequency inhibition after AT observed in BDNF^*Pax2*^-WT mice was not found in BDNF^*Pax2*^-KO animals (Fig. 7b, ****p < 0.0001). This indicates that after the onset of hearing, BDNF in lower brain parts increases the inhibitory strength of IC neuron that can be dropped after AT only when it has been increased before.

The stimulus-induced spike rates of IC neurons with middle and high CF in excitatory and noninhibitory regions were significantly elevated after AT in BDNF^*Pax2*^-WT in comparison to BDNF^*Pax2*^-KO mice (Fig. 7c). This is likely due to the drop in BDNF-driven inhibitory strength after AT in BDNF^*Pax2*^-WT but not in BDNF^*Pax2*^-KO mice (Fig. 7b). Importantly, these higher spike rates in BDNF^*Pax2*^-WTat animals occur without a corresponding increase in sound-evoked spike output as revealed through a smaller ratio between spikes in excitatory and noninhibitory areas in BDNF^*Pax2*^-WTat but not BDNF^*Pax2*^-KOat mice (Fig. 7d). When the spontaneous firing rates of IC neurons from poststimulus histograms for BBN were assessed after AT in BDNF^*Pax2*^-WT mice (Fig. 7e), there was a significant drop in the proportion of neurons with a spontaneous firing rate close to 0 (0–3 spikes/s) (32 % for WT to 61 % for KO, ***p < 0.001) and a shift of the distribution toward neurons with a higher spontaneous activity compared to BDNF^*Pax2*^-KOat animals (WT: Mdn = 3.8 to Mdn = 7.13, **p < 0.01; KO: Mdn = 4.13 to Mdn = 2.8, n.s. *p* > 0.5). These results underscore the loss of suppression of a BDNF-dependent firing rate. When spontaneous firing rates for low, middle, and high CF regions were analyzed separately, a significantly enhanced spontaneous firing rate for a CF >10 kHz after AT in BDNF^*Pax2*^-WT but not in BDNF^*Pax2*^-KO mice was observed (Fig. 7f). This finding strengthens the conclusion that BDNF may improve signal-to-noise ratio by increasing the inhibitory strength of neurons under basal conditions.

### Deletion of BDNF in BDNF^*Pax2*^-KO but not BDNF^*TrkC*^-KO Mice Reduces the Density of PV-Immunopositive Puncta in IC and AC Projecting Neurons

**Fig. 8:**
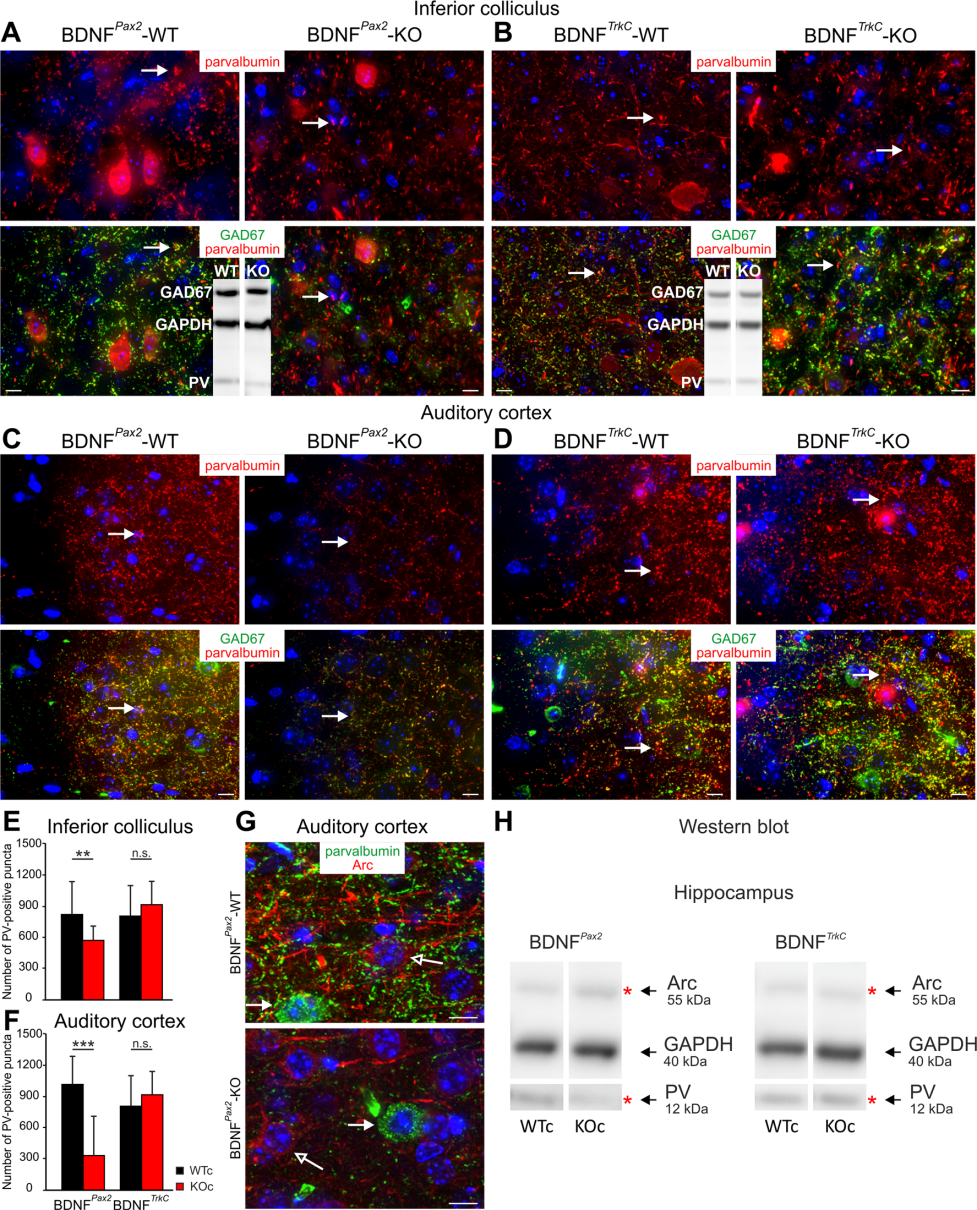
Immunohistochemistry of the inferior colliculus and auditory cortex of BDNF^*Pax2*^-KO and BDNF^*TrkC*^-KO mice. **a–d** Immunohistochemistry of the IC and AC of BDNF^*Pax2*^-KO (**a**, **c**) and BDNF^*TrkC*^-KO mice (**b**, **d**) immunolabeled with anti-parvalbumin (*red*) and anti-GAD67 (*green*). A reduction of PV- and GAD67-immunoreactive puncta in both IC (**a**) and AC (**c**) in BDNF^*Pax2*^-KO mice compared to BDNF^*Pax2*^-WT mice is observed. *Arrows* point to PV-immunoreactive puncta. No changes in PV and GAD67 expression are observed in the IC (**b**) and AC (**d**) of BDNF^*TrkC*^-KO mice compared to BDNF^*TrkC*^-WT mice. *Arrows* point to PV-immunoreactive puncta. Reduced PV levels in BDNF^*Pax2*^-KO but not BDNF^*TrkC*^-KO mice in the IC are confirmed by Western blot (**a**, **b** *insets*). **e**, **f** Quantification of PV-immunoreactive puncta in the IC (**e**) of BDNF^*Pax2*^-WT, BDNF^*Pax2*^-KO, BDNF^*TrkC*^-WT, and BDNF^*TrkC*^-KO mice (10–20 slices of 3–6 independent experiments of *n* = 3 animals) and in the AC (**f**) of BDNF^*Pax2*^-WT, BDNF^*Pax2*^-KO, BDNF^*TrkC*^-WT, and BDNF^*TrkC*^-KO mice (10–20 slices of 3–6 independent experiments of *n* = 3 animals). Two-way ANOVA with Bonferroni’s post hoc test (**p < 0.01, ***p < 0.001)*.* Data are presented as mean ± SD. **g** Immunohistochemistry of AC sections of BDNF^*Pax2*^-WT (*upper image*) and BDNF^*Pax2*^-KO (*lower image*) mice immunolabeled with anti-Arc (*red*) and anti-parvalbumin (*green*) antibodies. In BDNF^*Pax2*^-KO mice, Arc (*red*) expression is reduced. *Closed arrows* point to PV-positive cells. *Open arrows* indicate PV-immunoreactive puncta surrounding Arc-positive neurons. *Scale bars* = 10 μm. **h** Parvalbumin (*PV*) and Arc levels in the hippocampus of BDNF^*Pax2*^-WT and BDNF^*Pax2*^-KO mice and BDNF^*TrkC*^-WT and BDNF^*TrkC*^-KO mice, respectively, detected by Western blots (exemplarily for *n* = 3–5 mice). Note that the reduction of PV is associated to an increase of Arc in BDNF^*Pax2*^-KO but not BDNF^*TrkC*^-KO mice

In many projection neurons, changes in inhibitory conductances are assumed to require specialized GABA (γ-aminobutyric acid)_A_ receptors. Particularly, the differentiation of PV basket cells, a subpopulation of GABAergic neurons that regulate a critical period of plasticity in the cortex which depends on BDNF (for a review, see [54]), may play a role during the alteration of inhibitory strength in BDNF^*Pax2*^-KOs. Likewise, elevated auditory startle amplitude and latencies in PV^−/−^ mice suggested a specific role of PV basket cells for inhibitory circuits in auditory processing [55]. To investigate a role of PV for the observed changes in inhibitory strength in BDNF^*Pax2*^-KO mice, we used antibodies for PV [56, 57] and antibodies for the 67-kDa isoform of GABAergic interneurons. We found that the intensity and number of PV-immunopositive puncta, but not the number of PV-immunopositive somata or GAD67-immunopositive puncta (not shown), are decreased in the IC and AC of BDNF^*Pax2*^-KO (Fig. 8a, c) but not in BDNF^*TrkC*^-KO mice (Fig. 8b, d). Reduced levels of PV expression could also be confirmed by Western blots (Fig. 8a, b, inset). Quantification of the intensity of labeling revealed a significantly reduced number and intensity of PV-positive puncta (Fig. 8e, 10–20 slices of 3–6 independent experiments of *n* = 3 animals) in BDNF^*Pax2*^-KO mice. Also in the hippocampus, PV levels were reduced in BDNF^*Pax2*^-KO but not in BDNF^*TrkC*^-KO mice, as exemplarily shown by Western blots (Fig. 8h). Interestingly, the reduction of PV levels in BDNF^*Pax2*^-KO mice was associated with elevated levels of the immediate early gene Arc (activity-regulated cytoskeletal protein), as exemplarily shown for the hippocampus (Fig. 8h, *n* = 3–5). Arc is mobilized in glutamatergic neurons following, e.g., long-term potentiating (LTP)-like activity [58, 59]. As BDNF^*Pax2*^-KO mice do not lack BDNF in the AC and hippocampus (Fig. 1c, e), future studies are required to investigate a potential developmental downregulation of Arc levels mediated by BDNF-dependent increases in inhibitory strength following onset of sensory function.

**Fig. 9:**
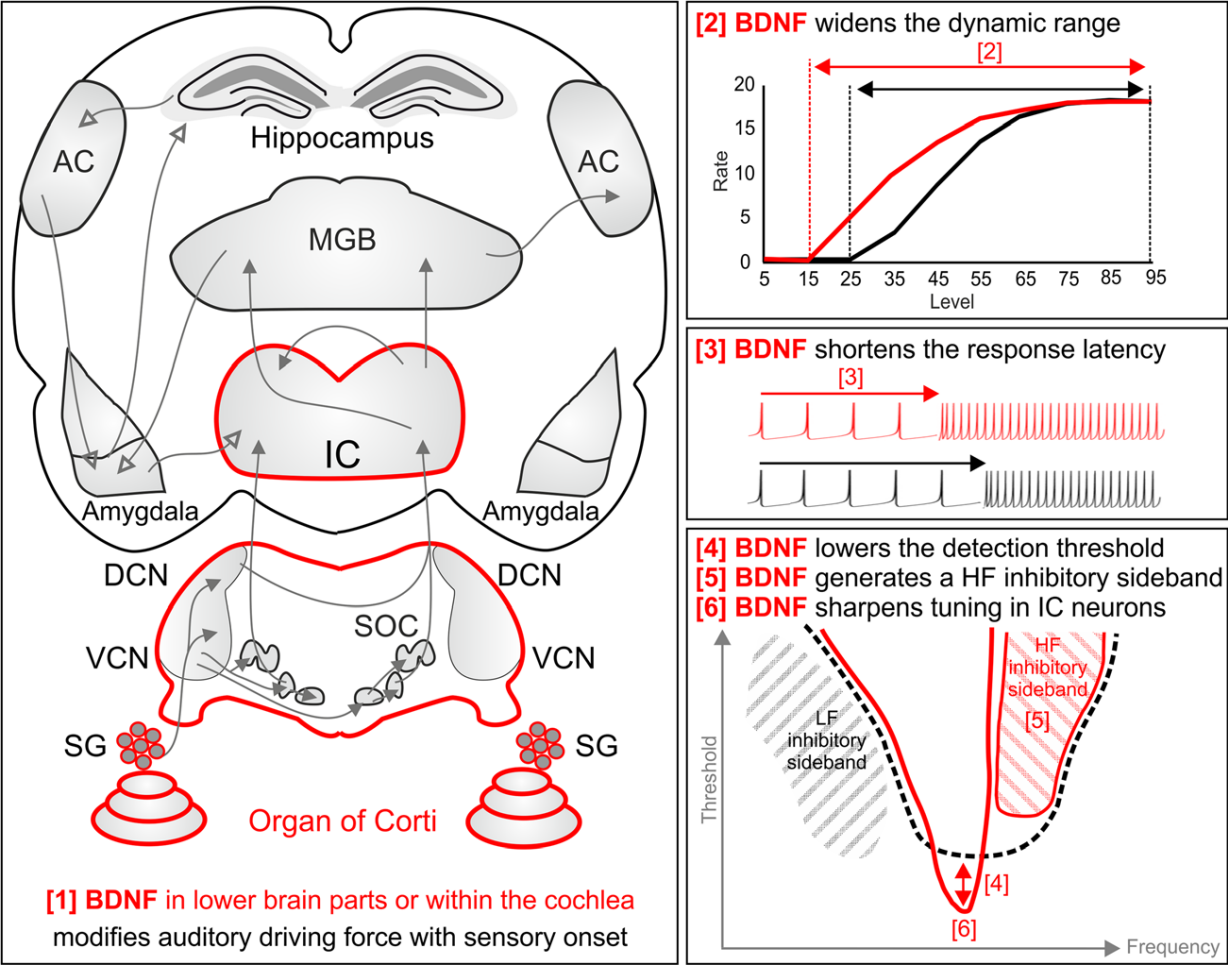
Schematic summary of the results that illustrates our hypothesis. An auditory driving force which is modified with hearing onset and depends on BDNF in the lower parts of the auditory system or within the cochlea [*1*] widens the dynamic range above which spike rates can be detected [*2*]. This is achieved upon shortening of the response latency [*3*], leading to lowering of the detection threshold [*4*] through generation of a high-frequency inhibitory sideband [*5*] in, e.g., very narrowly tuned IC neurons [*6*]. Through these events, the probability to detect a spike above the noise floor may be improved. This BDNF activity on auditory fibers in high-frequency cochlear turns can be lost after acoustic trauma. In this case, spontaneous spike rates in central auditory pathways are elevated (hyperactivity). As a consequence, due to loss of basal inhibitory strength within the ascending circuit of affected frequency regions, the capacity to adapt to sensory deprivation might be reduced. *HF* high frequency, *IC* inferior colliculus, *IHC* inner hair cell, *LF* low frequency

In conclusion and as outlined in Fig. 9, the data in the present study suggest that BDNF in the lower parts of the auditory system or within the cochlea (and not in the upper central auditory system) (Fig. 9 [1]) alters the afferent driving force for sound processing toward a wider dynamic range to which IC neurons can respond to sound intensities (Fig. 9 [2]). This is possibly mediated by generation and maintenance of increased inhibitory PV contacts with the auditory brain network that leads to enhanced spike probability in response to sensory stimulation. As shown for IC neurons, the BDNF-modified afferent driving force shortens response latency (Fig. 9 [3]) and lowers the CF detection threshold in IC neurons (Fig. 9 [4]) through the generation of a high-frequency inhibitory sideband (Fig. 9 [5]) in, e.g., very narrowly tuned IC neurons (Fig. 9 [6]). This suggests that the auditory driving force which depends on BDNF in the lower parts of the auditory CNS or within the cochlea may improve fidelity through enhancing the inhibitory strength within the auditory-specific network. When this driving force is lost after auditory nerve injury, such as following acoustic trauma, as shown here for traumatized BDNF^*Pax2*^-WT animals, the auditory-specific network may lose its normal signal-to-noise ratio within the affected frequency range.

## Discussion

The present study supports the hypothesis that the maturation of auditory nerve activity depends on BDNF in the lower parts of the auditory CNS or within the cochlea and is required to improve the fidelity of sound-induced responses after the onset of hearing. Improved auditory fidelity under control of this BDNF-dependent process is most likely achieved through enhancement of basal inhibitory circuits in the ascending auditory pathway. In the mature auditory organ, this BDNF-dependent driving force can be lost during, e.g., cochlear injury, leading to an elevated spontaneous firing rate, independently of a corresponding increase in sensory-evoked spikes.

### Peripheral but not Central BDNF Drives Changes in Sound Processing

We show here that BDNF present in the lower parts of the auditory CNS or the peripheral end organ improves the threshold for sound and the dynamic range of sound responses. In both BDNF^*Pax2*^-KO [25] and BDNF^*TrkC*^-KO animals (present study), BDNF is deleted in SGNs and in the IC, suggesting that alterations of BDNF activities in these compartments are unlikely to be responsible for the differences between sound-induced ABR wave I and IV responses in these mice. As BDNF^*Pax2*^-KO mice do not have a BDNF deletion in neither the AC, VCN, or olivary complex [present study] [25], descending central feedback loops are also unlikely to be involved. The observed hearing phenotype in BDNF^*Pax2*^-KO mice may therefore be linked to a Pax2-driven BDNF deletion in the lower parts of the auditory CNS or the cochlea. In the cochlea, BDNF is expressed postnatally in phalangeal, SGN, or satellite cells [22, 25, 50, 60]. Given that (i) BDNF deletion in phalangeal cells does not affect hearing thresholds [23], (ii) BDNF deletion in SGNs and in a minor number of supporting cells as observed in BDNF^*TrkC*^-KO mice does not lead to an auditory phenotype, and (iii) BDNF expression in inhibitory interneurons has been excluded [52, 61], the loss of BDNF expression in glial cells may be considered as being responsible for the observed phenotype in BDNF^*Pax2*^-KO animals.

### Peripheral BDNF Improves Amplitudes of Spreading Sound-Induced Activity

In our previous study, reduced exocytosis in otherwise mature IHCs in high-frequency cochlear turns of 2–3-week-old BDNF^*Pax2*^-KO mice [25] pointed out a critical role of BDNF for pre- and postsynaptic maturation of IHCs. The alterations in ABR waves I and IV found in the present study in BDNF^*Pax2*^-KO but not BDNF^*TrkC*^-KO mice indicate that BDNF activities that are carried out independently of higher auditory brain regions improve sound sensitivity at least up to the level of the IC. We assume that in BDNF^*Pax2*^-KO mice the decrease of ABR wave I and IV amplitude observed before AT and its reduced decline after AT occurs independently of the electromechanical properties of OHCs for the following reasons: (i) within the range of the stimulus levels in which ABR functions were quantified, no difference in DPOAEs, used as a measurement for mechanical properties of OHCs, was found; (ii) changes of suprathreshold responses after AT are seen in BDNF^*Pax2*^-KO mice, but no differences are observed in DPOAE amplitudes after AT; and (iii) BDNF is not deleted in the olivocochlear brainstem nuclei that are projection fields of efferent fibers toward OHCs [25]. BDNF is therefore more likely to improve ABR amplitudes through its influence on IHC exocytosis and/or auditory fiber sensitivity for low sound thresholds. In this context, it is important to consider that low-threshold fiber characteristics develop only after hearing onset [62, 63]. Also in the visual system, a role of retinal BDNF for improved retinal and visual acuity has been suggested [64]. Accordingly, an antisense-based inhibition of BDNF in the retina prevented an improvement of retinal stripe segregation induced by environmental enrichment as well as cortical acuity, even prior to eye opening [16, 17, 64]. To date, conditional deletion of retinal BDNF has not been performed, and it is therefore elusive if it improves the baseline of visual sensitivity independently of environmental enrichment, similar to the present findings.

### BDNF Drives Increased Inhibitory Strength Along the Auditory Pathway

The current findings suggest that BDNF-dependent effects on auditory nerve activity are responsible not only for improving the ABR amplitude size of high-frequency sound responses spreading along the ascending pathway, but also for expanding the range of CF thresholds in these high-frequency CF regions. The affected CF regions >10 kHz span areas (15–20 kHz) where murine behavioral auditory thresholds are at their lowest level [65]. We thus have to consider the improved CF thresholds, short latencies, and the increased strength of inhibitory sidebands of sharply tuned IC neurons in BDNF^*Pax2*^-WT versus BDNF^*Pax2*^-KO animals in the context of an enhanced auditory fidelity in behaviorally relevant frequency regions. The IC neurons that show altered tuning characteristics depending on the presence of BDNF are reminiscent of type II [65] and type I [66] class IC neurons. Both IC cell types predominate in high-frequency regions and are characterized by their short latency, narrow and sharp tuning, low-frequency border with a shallow slope, and high-frequency border with a steep slope [65, 66]. It thus may be considered that these neurons are shaped under the control of BDNF activities at hearing onset.

In BDNF^*Pax2*^-KO animals, the proportion of neurons with an elevated spontaneous firing rate is slightly, however not yet significantly, increased. After AT, however, a significant drop in neurons with a lower spontaneous firing rate and an increase in neurons with a higher spontaneous firing rate in BDNF^*Pax2*^-WT mice compared to BDNF^*Pax2*^-KO animals support a loss of the BDNF-dependent rate of firing suppression after auditory nerve injury. Reminiscent to this finding, a reduced rate of firing suppression that leads to an increase of the spontaneous firing rate without a stimulus-evoked spike output was described after a blockade of tonic inhibition in granule cells of the cerebellar cortex [67]. It is currently assumed that tonic inhibition suppresses spontaneous activity through reduction of the neuronal input resistance and membrane time constants, thereby improving stimulus discrimination above noise [68]. The ability of tonic inhibition to change conductances in many neurons is assumed to require perisynaptic and extrasynaptic δ subunit-containing GABA_A_ receptors, which are likely to be activated through fast-spiking, PV-expressing, and soma-inhibiting interneurons [69]. PV-expressing interneurons play a crucial role for hippocampal microcircuit formation and plasticity changes [70]. The significant loss of density of PV-immunopositive puncta in the IC and AC and the first evidence for declined PV levels associated with elevated Arc levels in the hippocampus of BDNF^*Pax2*^-KO but not in BDNF^*TrkC*^-KO mice may suggest that a BDNF-modified driving force for auditory processing improves the baseline for mature circuit formation and plasticity changes along the entire ascending auditory network. This finding, moreover, suggests that the altered brain network activity shaped under the influence of BDNF in the lower parts of the auditory CNS or within the cochlea is a prerequisite for cortical maturation processes that occur with sensory experience [71–73]. The influence of dendritogenesis of PV-containing interneurons in the olfactory bulb [12], the auditory [8], visual [17, 74], and somatosensory cortex [11] on improved spectral and temporal cortical response properties [71–73] should be reconsidered in the context of these findings.

### Peripheral BDNF Improves Sound Fidelity but Carries the Risk to Enhance Central Noise Following Its Loss After Injury

We observed here that suprathreshold ABR wave IV and CF threshold in IC neurons after AT were persistently more elevated in BDNF^*Pax2*^-WT than in BDNF^*Pax2*^-KO mice. This may be explained by a loss of auditory fiber characteristics that depend on BDNF and that are essential to maintain tonic inhibitory strength in the ascending pathways. As a result, spontaneous firing rates may be elevated within the affected frequency range without leading to sound-induced output as shown here in BDNF^*Pax2*^-WT but not in BDNF^*Pax2*^-KO mice.

A persistent reduction of suprathreshold amplitudes at the level of the IC has also been observed in traumatized rats with behaviorally tested tinnitus [38, 60] indicating that lost auditory nerve fiber activities that are typically maintained by BDNF in the lower parts of the auditory CNS or within the cochlea may be causally related to “phantom” noise. Previous observations that describe elevated intensity discrimination thresholds in tinnitus subjects with normal audiograms [75] may be re-examined in the context of a loss of signal-to-noise ratio in those frequency bands in which the BDNF-dependent driving force is lost. When the BDNF-dependent driving force is lost, the baseline levels required to allow the development of a normal adaptation of central brain responses after cochlear injury may be lost. In view of the current findings, a BDNF-dependent auditory driving force may thus be the prerequisite for adaptive responses and central gain [76] after sensory deprivation [74, 77, 78]. Central maladaptive hyperactivities in various brain disorders including pain, Alzheimer, or epilepsy [79–81] and their enigmatic relationship to BDNF may be revisited in the context of the current findings [20, 21, 80]. For these disorders, a critical loss of the BDNF-dependent driving force should be taken into account.

## Abbreviations

ABR: Auditory brainstem response
AC: Auditory cortex
Arc: Activity-regulated cytoskeletal protein
AT: Acoustic trauma
BBN: Broadband noise
BDNF: Brain-derived nerve growth factor
CF: Characteristic frequency
CN: Cochlear nucleus
DCN: Dorsal cochlear nucleus
DPOAE: Distortion product of the otoacoustic emission
IC: Inferior colliculus
IHC: Inner hair cell
KO: Knockout
mFSL: Minimum first-spike latency
OHC: Outer hair cells
PV: Parvalbumin
RIF: Rate-intensity function
ROSA26R: ROSA26 reporter mouse
SGN: Spiral ganglion neuron
SPL: Sound pressure level
VCN: Ventral cochlear nucleus
WT: Wild type
β-gal: β-Galactosidase
